# The posterior capsular central amygdala showing synaptic coactivation with nociplastic pain-associated parabrachial neurons in mice

**DOI:** 10.1016/j.isci.2025.113001

**Published:** 2025-06-25

**Authors:** Takao Okuda, Sawako Uchiyama, Naoko Sato, Yae K. Sugimura, Yukari Takahashi, Makoto Tsuda, Fusao Kato

**Affiliations:** 1 Department of Neuroscience, The Jikei University School of Medicine, Minato-ku, Tokyo 105-8461, Japan; 2 Center for Neuroscience of Pain, The Jikei University School of Medicine, Minato-ku, Tokyo 105-8461, Japan; 3 Department of Molecular and System Pharmacology, Graduate School of Pharmaceutical Sciences, Kyushu University, Fukuoka 812-8582, Japan

**Keywords:** Natural sciences, Biological sciences, Neuroscience, Systems neuroscience, Sensory neuroscience

## Abstract

Projections from the external lateral parabrachial nucleus (elPB) to the central amygdala (CeA) are a key pathway for nociceptive signals and play a crucial role in establishing nociplastic pain sensitization, a state of heightened pain without nociceptor activation or nerve injury. To investigate their roles in nociplastic pain, we aimed to analyze how pain-activated elPB neurons transmit information to pain-activated CeA neurons. Using transgenic TRAP2 mice that underwent transient localized inflammation, we selectively expressed marker proteins, light-sensitive channels, and chemogenetic receptors in pain-activated neurons. We found that the pain-activated (“nociTRAPed”) neurons in the elPB project extensively to the CeA, particularly to the caudal half of the capsular CeA (defined as “posterior CeC” or pCeC), where they form robust functional connections with nociTRAPed pCeC neurons, promoting nociplastic sensitization. We propose that the pCeC serves as the site of direct co-activation with pain-activated elPB neurons, translating peripheral nociceptive information into CeA excitation.

## Highlights

• We expressed ChR2-YFP in neurons activated during inflammation in fosTRAP2 mice
• NociTRAPed parabrachial neurons (elPB) projected widely, most densely to the CeA
• NociTRAPed elPB neurons form robust synaptic connections with those in the pCeC
• Excitation of the nociTRAPed CeA neurons reproduced bilateral hindpaw sensitization

## Graphical abstract

**Figure:**
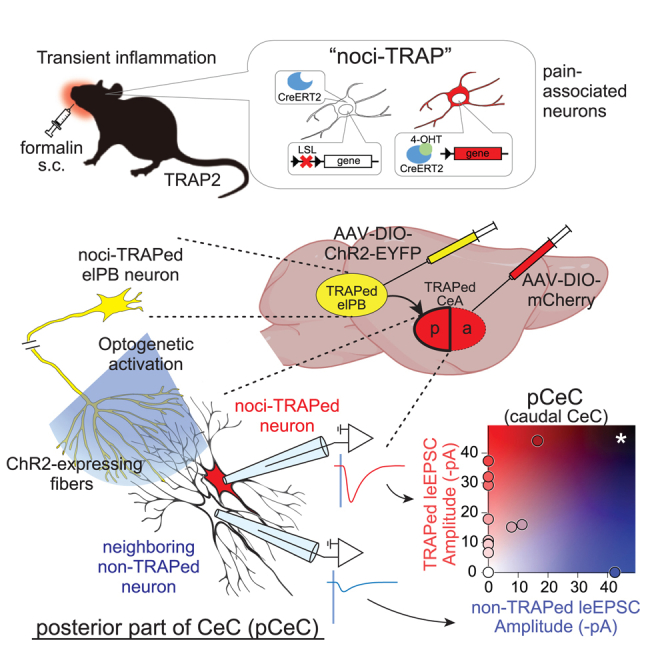

## Introduction

Chronic pain is a global health problem affecting more than 20% of the population. In addition to the well-established nociceptive and neuropathic pain, a more recently proposed mechanistic descriptor, “nociplastic pain”,^1^ and the novel clinical classification, “chronic primary pain”,^2^ have garnered worldwide attention. This is due to their ability to explain pain occurring without clear evidence of actual or potential tissue damage at the site of pain-associated sensitization. The central concept of nociplastic or primary chronic pain is that altered activities in the brain networks underlying pain signaling form the basis for various forms of pain without identifiable causes, as seen in conditions, such as fibromyalgia, chronic widespread pain, chronic temporomandibular disorders, and irritable bowel syndrome.^3^

Indeed, various forms of brain plasticity have been observed in multiple brain regions associated with chronic pain in human patients,^4^^,^^5^ as well as in rodent models of acute-to-chronic pain.^6^^,^^7^^,^^8^^,^^9^^,^^10^^,^^11^ Among the diverse brain networks involved in these pain-associated plastic changes, the amygdala—particularly the central amygdala (CeA)—has attracted significant interest. In human patients, sustained or heightened amygdala activity has been shown to predict recovery-resistant back pain,^12^ and the activity of the parabrachial-to-amygdala pathway is a key determinant of nociceptive aversive sensations in humans.^13^

In rodents, the CeA receives nociceptive information from the external lateral parabrachial nucleus (elPB), which itself receives inputs from nociception-specific neurons in the superficial layer of the lumbar dorsal horn (DH) and the caudal part of the spinal trigeminal nucleus (Sp5c).^14^ Additionally, the elPB receives direct input from many trigeminal C-fibers, directly relaying nociceptive information from the face and head to the CeA.^15^ In various models of sustained or late-onset post-formalin sensitization, increased expression of phosphorylated extracellular signal-regulated kinase (pERK) and c-Fos in the CeA,^6^^,^^10^^,^^16^ along with robust synaptic potentiation of transmission from the elPB to the CeA,^17^^,^^18^^,^^19^^,^^20^ and heightened excitability of CeA neurons,^21^ have been documented. We have shown that formalin injection into the left plantar surface induces tactile sensitization in both the ipsilateral and contralateral hind paws, accompanied by enhanced synaptic transmission from the elPB to the right CeA. This potentiation and the ectopic sensitization are absent in mice lacking calcitonin gene-related peptide (CGRP).^19^ These findings suggest that pain-associated activation of the elPB-CeA pathway may contribute to ectopic tactile sensitization in regions distant from the site of injury or inflammation. Moreover, experimental activation of CeA neurons using optogenetic or chemogenetic techniques can actively induce sensitization across various regions of the body.^22^^,^^23^^,^^24^ These lines of evidence indicate that co-activation of elPB and CeA neurons plays a crucial role in central sensitization in numerous persistent pain models.^23^ Based on this evidence, it is commonly hypothesized that nociceptive elPB neurons, which receive inputs from trigeminal or spinal nociceptive neurons, directly excite nociceptive CeA neurons, leading to central sensitization.

Nevertheless, some recent evidence challenges this straightforward interpretation. For example, in formalin-induced sensitization models, the expression of c-Fos in the CeA, particularly in the capsular part (CeC) where many elPB fibers terminate, showed only a weak correlation with c-Fos expression in the elPB of the same animal.^10^ This suggests that the activation of CeC neurons is not simply a synaptic consequence of excitatory inputs from the elPB. Indeed, the elPB-CeA projection conveys not only nociceptive information but also signals related to other modalities, such as inflammation-associated malaise, appetite, and body temperature.^24^^,^^25^ This implies that excitatory inputs from the elPB are not functionally homogeneous. Thus, the precise manner in which nociceptive elPB neurons project to and excite nociceptive CeA neurons remains a key question in understanding the role of the elPB-CeA pathway in central sensitization. However, this is a difficult question to address due to the challenges in selectively stimulating and recording from neurons with a history of activation in persistent pain models.

To tackle this issue, we utilized a transgenic mouse line, TRAP2 mice,^26^^,^^27^ which allows for the selective expression of exogenous proteins in neurons with a time-delimited history of c-Fos expression. Using this technique, we successfully visualized nociceptive elPB and CeC neurons (i.e., “nociTRAPed” neurons) and selectively stimulated the fibers of nociTRAPed elPB neurons while recording from CeA neurons. We compared postsynaptic responses in neighboring pairs of CeA neurons—one nociTRAPed and one non-nociTRAPed—evoked by light stimulation of nociTRAPed elPB fibers within the CeA slice. Our findings indicate that in the caudal half of the CeC (posterior CeC, pCeC), fibers from nociTRAPed elPB neurons form robust excitatory synaptic connections with nociTRAPed pCeC neurons at a higher rate. This suggests a specific role for the pCeC in relaying nociceptive information from peripheral nociceptive systems to CeA excitation through the parabrachial relay.

## Results

The development of chronic nociplastic pain involves a transition from acute pain to a long-lasting painful state. This persistent state is marked by abnormal pain-related behaviors, which emerge after the initial acute pain responses caused by tissue injury or inflammation subside. In the post-formalin late-onset sensitization model,^23^^,^^28^ acute pain behaviors, like face rubbing, diminish within 1 h, and symptoms of chronic pain, such as widespread abnormal sensitization, begin to appear around 3 h after formalin injection.^23^ This transition is accompanied by the activation of specific molecules in the right CeA, which are involved in subsequent neuronal plasticity, such as c-fos and phosphorylated extracellular signal-regulated kinase (ERK).^6^^,^^10^^,^^16^ Using these time-dependent properties, we sought to genetically “mark” the cells activated in this process of the shift toward chronic pain using a fosTRAP mouse.

### Upper lip formalin injection resulted in robust expression of tdTomato in Fos-TRAP2::Ai14 mice

**Figure 1:**
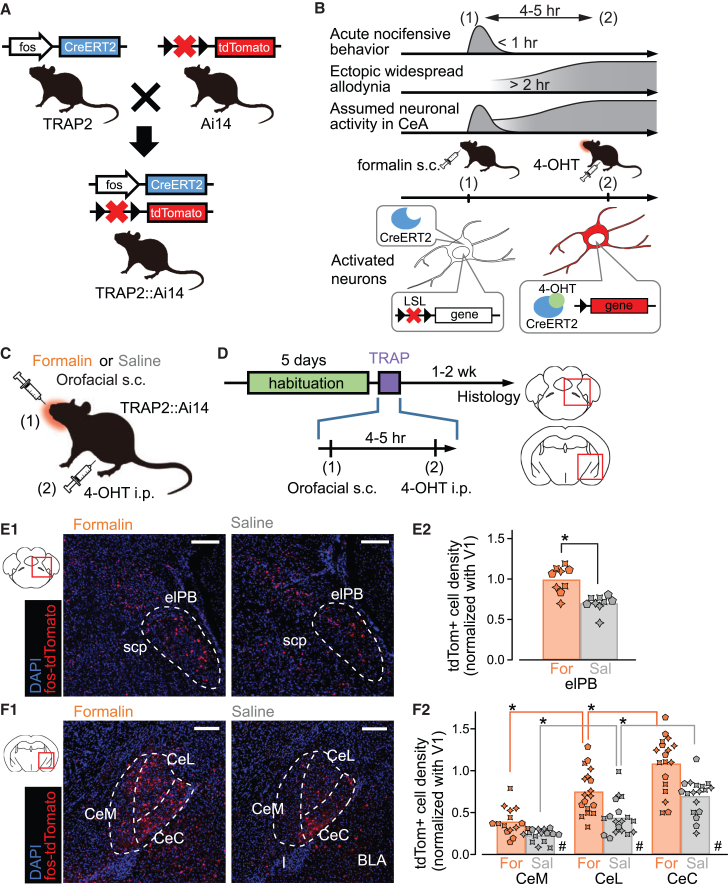
Principles and protocols for “nociTRAP-ing” with orofacial formalin injection and visualization of nociTRAPed neurons in the elPB and CeA (A) Schematic of the mating strategy for the transgenic mice. Fos-2A-iCre (TRAP2) mice were mated with Ai14-LSL-tdTomato reporter mice to generate TRAP2::Ai14 mice. Red crosses and black triangles indicate the floxed stop codon (*loxP* stop *loxP* LSL). (B) Schematic timeline of activity-dependent molecular expression using the fosTRAP method in the post-formalin late-onset sensitization model. The top three conceptual waves illustrate changes in acute pain behavior, ectopic widespread allodynia, and assumed neuronal activity in the CeA during formalin-induced inflammatory pain. The timescale at the bottom indicates the timing of orofacial formalin s.c. injection (1) and 4-OHT i.p. administration (2). 4-OHT was administered 4–5 h after the orofacial formalin injection. The bottom panel illustrates how the tdTomato is expressed in the activated neurons. Immediately after formalin injection into the unilateral upper lip, CreERT2 protein is expressed with c-fos protein in activated neurons (1). Injected 4-OHT activates CreERT2 and promotes its translocation into the nucleus. Neurons with prolonged activation are FosTRAPed. (C) Schematic of the FosTRAP experiment in the post-formalin late-onset sensitization model. Formalin or saline s.c. injection into the right upper lip (1), followed by 4-OHT i.p. administration (2). (D) Experimental procedure of the FosTRAP technique. Mice were habituated for 5 days before fosTRAP. Unless stated otherwise, 4-OHT was administered 4–5 h after formalin or saline injection. (E) Quantification of tdTomato+ cells in the elPB. (E1): representative fluorescence images of brain sections containing the right lateral parabrachal nucleus from formalin- (left) and saline- (right) injected mice (approximately −5.2 mm from bregma). Dashed lines show the right elPB. Scale bars: 200 μm. (E2): normalized density of Tomato+ cells. Values were calculated from three coronal sections from 3 mice in each group. The same markers indicate values from the same mice. ∗ indicates statistically significant differences between treatments (Mann-Whitney U test; U = 72, *p* = 0.0012). (F) Quantification of tdTomato+ cells in the CeA. (F1): representative fluorescence images of brain sections containing the right CeA from formalin- (left) and saline- (right) injected mice (approximately −1.4 mm from bregma). Dashed lines show the right CeA subregions: CeC, CeL, and CeM (capsular, lateral, and medial parts of the central amygdala); BLA, basolateral amygdala; I, intercalated nuclei of the amygdala. Scale bars: 200 μm. (F2): normalized density of tdTomato+ cells. Values were calculated from six coronal sections from 3 mice in each group. The same markers indicate values from the same mice. # and ∗ indicate statistically significant differences between treatments and between CeA subnuclei, respectively (Kruskal-Wallis test; H(5) = 64.503, #, *p* < 0.0013, *p* < 0.0010, *p* < 0.0013 formalin vs. saline in the CeM, CeL, CeC, respectively; ∗, *p* < 0.001 and = 0.0012, CeM vs. CeL in formalin and saline group, respectively; *p* = 0.0036 and 0.0012, CeL vs. CeC in formalin and saline group; *p* < 0.001 and *p* < 0.001, CeM vs. CeC in formalin and saline group).

First, we examined how neurons in the subnuclei of the elPB and the CeA were activated 4–5 h after upper lip formalin injection. To visualize the neurons that expressed c-fos and cre recombinase, we used FosTRAP2 mice crossed with Ai14 mice, which express the tdTomato protein. The principles and procedures of this TRAPing are shown in Figures 1A–1D. Unless otherwise stated, we injected 4-OHT (50 mg/kg i.p.) at 4–5 h after formalin injection to the right upper lip. With these procedures, we could visualize cells with a history of fos expression using the fluorescence signal of tdTomato as a proxy (Figures 1E1 and 1F1, respectively). In this article, we refer to these neurons as “nociTRAPed,” which had been activated at 3–5 h after the 4-OHT injection.^26^^,^^29^ We found more nociTRAPed neurons in the elPB and the CeA, particularly in the CeC and lateral CeA (CeL) of formalin-injected mice compared to those receiving saline (Figure 1F1).

Preliminary experiments using hind paw injection of formalin followed by i.p. injection of 4-OHT at 2 h and 5 h post-formalin or post-saline and 4-OHT injection without precedent formalin injection (including the needle piercing into the skin) indicated that hind paw injection of saline at 5 h before 4-OHT injection, but not much at 2 h, resulted in similarly small number of fosTRAPed neurons in the CeA to the fosTRAPing without noxious stimulation (Figures S1A–S1C). This 5-h interval also resulted in high contrast in the number of fosTRAPed CeA neurons between saline and formalin injected mice. Also considering the metabolic time course of 4-OHT and gene expression time course of c-fos after cre recombination (Figure 1B), we used the 5-h interval between upper-lip formalin injection (s.c.) and 4-OHT injection (i.p.) in the fosTRAPing experiments described in this paper.

We then statistically compared the fraction of nociTRAPed cells between different CeA subregions and experimental maneuvers. To account for differences in non-specific activation levels between different mice, we normalized the number of TRAPed cells in the elPB by the number of TRAPed cells in the bilateral primary visual area (V1) of the same mouse, as the activation of visual cortex neurons would not be and indeed was not detectably affected by formalin injection (Figures S1D and S1E). In the elPB and the three CeA subnuclei, the number of TRAPed cells was significantly higher in formalin-treated groups than in saline-treated groups (Mann-Whitney U test for the elPB, ∗ in Figure 1E2, and multiple comparisons using the Kruskal-Wallis test for the medial CeA (CeM), CeL, and CeC, # in Figure 1F2). In addition, we found a significantly greater number of TRAPed cells in the CeC, followed by the CeL, with the smallest number in the CeM in both saline- and formalin-treated animals (∗ in Figure 1F2). This significantly greater number of nociTRAPed cells in the elPB and CeA, particularly in the CeC after orofacial formalin treatment, is consistent with our previous results showing a significant increase in c-fos expressing cells in the elPB and CeA in rats 3 h after formalin injection.^10^

### The TRAPed elPB neurons showed prominent projections to the CeA

**Figure 2:**
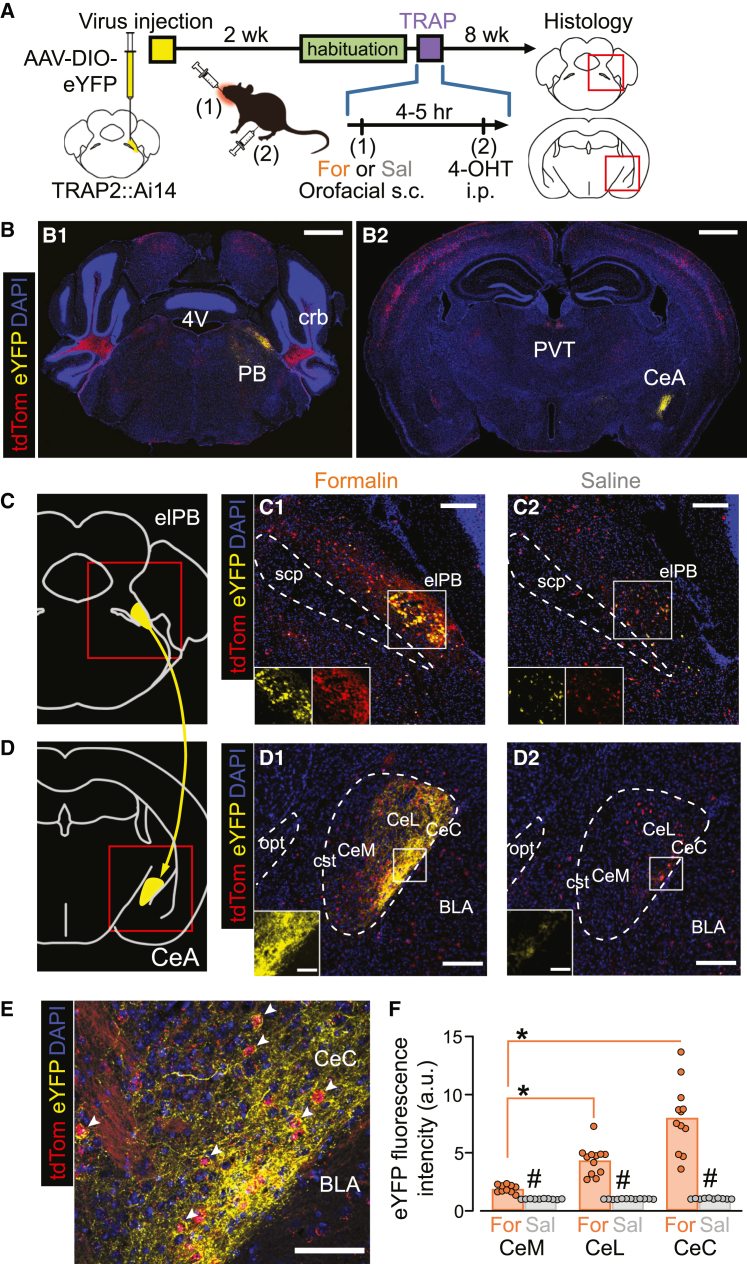
Visualization and evaluation of nociTRAPed elPB axonal projections to the CeA (A) Experimental procedure for visualizing the projections of TRAPed elPB neurons to the CeA. Virus injection was performed 2 weeks before fosTRAP, and brain tissues were collected 8 weeks after fosTRAP to allow for sufficient protein expression in long-projecting axons. (B) Representative coronal brain sections showing intense YFP signals at the elPB (virus injection site [approximately −5.4 mm from bregma]), (B1) and in the CeA (axon target [approximately −1.2 mm from bregma]), (B2). The scale in (B1 and B2) is the same. PB, parabrachial nucleus; crb, cerebellum; 4V, 4th ventricle; PVT, paraventricular thalamic nucleus. Scale bars: 500 μm. (C and D) Schematics of CeA-projecting elPB neurons (leftmost in C and D) and representative images of the elPB and CeA in the formalin group (C1 [approximately −5.4 mm from bregma]), D1 (approximately −1.4 mm from bregma]) and the saline group (C2 and D2). The lower left inset images in (C1) and (C2) show fluorescent signals in the rectangular areas of the elPB (yellow, eYFP; red, tdTomato). The lower left inset images in (D1) and (D2) show the eYFP signal in the rectangular area of the CeA. Scale bars: 200 μm (in C1, C2, D1, and D2); 50 μm (in the lower left inset of D1 and D2). scp, superior cerebellar peduncle; elPB, external lateral parabrachial nucleus; opt, optic tract; cst, commissural stria terminalis; CeC, CeL, CeM (capsular, lateral, and medial parts of the central amygdala, respectively); BLA, basolateral amygdala. (E) High-magnification confocal microscope image of the CeA showing eYFP in fibers originating from nociTRAPed elPB neurons (yellow) and nociTRAPed neurons in the CeA (red). White arrowheads indicate tdTomato-positive somas surrounded by eYFP-positive fibers. Approximately −1.4 mm from bregma. Scale bar: 50 μm. (F) Mean relative fluorescence intensity of eYFP signals in each subregion at each coronal level in both groups. Values were calculated from six coronal sections from 2 mice in each group. # and ∗ indicate statistically significant differences between treatments and between CeA subnuclei, respectively (Kruskal-Wallis test; H(5) = 57.536, #, *p* < 0.001, *p* < 0.001, *p* < 0.001 formalin vs. saline in the CeM, CeL, and CeC, respectively; ∗, *p* < 0.001 and *p* > 0.99, CeM vs. CeL in formalin and saline group, respectively; *p* = 0.003 and 0.17, CeL vs. CeC in formalin and saline group; *p* < 0.001 and = 0.11, CeM vs. CeC in formalin and saline group).

How do these nociTRAPed elPB neurons project? To visualize their axons, we injected an adeno-associated virus (AAV) encoding Cre-dependent eYFP (AAV-DIO-eYFP) into the right elPB of TRAP2::Ai14 mice two weeks before nociTRAPing (Figure 2). Eight weeks after the nociTRAPing, we observed dense eYFP-positive axons projecting to the ipsilateral CeA, along with tdTomato-labeled somata in the target area (Figure 2B2).

Although less dense than the projections to the CeA, eYFP-positive fibers were also detected in several brain regions implicated in pain-associated signaling, including the dorsal periaqueductal gray (Figures S2A1, S2B1, and S2C1), the paraventricular thalamus (PVT; Figures S3A1, S3B1, and S3C1), the anterior PVT (∗ in Figure S3D1), the median preoptic nucleus (∗∗ in Figure S3D1), the lateral hypothalamus (Figures S3A2, S3B2, and S3C2), the para-subthalamic nucleus (Figures S3B2), the posterior portion of the basolateral amygdala (BLA; Figure S2C5, with no fibers detected at more anterior BLA levels), the bed nucleus of the stria terminalis (BNST; Figure S3F2), and the insular cortex (Figures S3E5 and S3F5). These projections were predominantly ipsilateral; however, a smaller number of eYFP-expressing fibers were also observed in the contralateral PVT (Figures S3B1 and S3C1) and in the contralateral CeA, particularly in the capsular part (Figure S3B5).

To confirm that the axonal eYFP expression depends on neuronal excitation in the elPB following transient formalin-induced inflammation, we compared the eYFP-positive fibers in the CeA between formalin- and saline-injected groups. In both formalin- and saline-TRAPed mice, the eYFP signals (left inset at the bottom of Figures 2C1 and 2C2) mostly overlapped with those of the tdTomato signals (right inset at the bottom of Figures 2C1 and 2C2), indicating that the eYFP signals in the CeA represent fibers arising from nociTRAPed elPB neurons. Within the subregions of the CeA, eYFP-positive fibers were richest in the CeC subregion, sparser in the CeL, and almost invisible in the CeM (Figure 2E). We observed many neurons in the CeC expressing tdTomato in the soma, and occasionally, some of these were surrounded by eYFP-expressing fibers (white arrowheads in Figure 2E), reminiscent of the “basket synapses” previously reported in elPB-CeC connections.^30^^,^^31^ We quantified the subregion-dependent eYFP fluorescence intensity in brain sections from formalin-TRAPed mice (6 sections each from 2 mice) and saline-TRAPed mice (6 sections each from 2 mice) (Figure 2F). In addition to the significantly higher fluorescent intensity in formalin-TRAPed mice compared to saline-TRAPed mice (# in Figure 2F), the fluorescent intensity was significantly higher in the CeC than in the CeM, and higher in the CeL than in the CeM (∗ in Figure 2F). This subregion-dependent difference was not observed in the saline-treated groups (Figure 2F). These results are consistent with previous reports showing that the CeC receives excitatory inputs from the elPB^20^^,^^32^^,^^33^^,^^34^^,^^35^^,^^36^ and exhibits the most extensive c-fos expression after formalin injection in rats.^10^

### Rostrocaudal distribution of intra-CeA fibers from TRAPed elPB neurons and TRAPed CeA neurons

**Figure 3:**
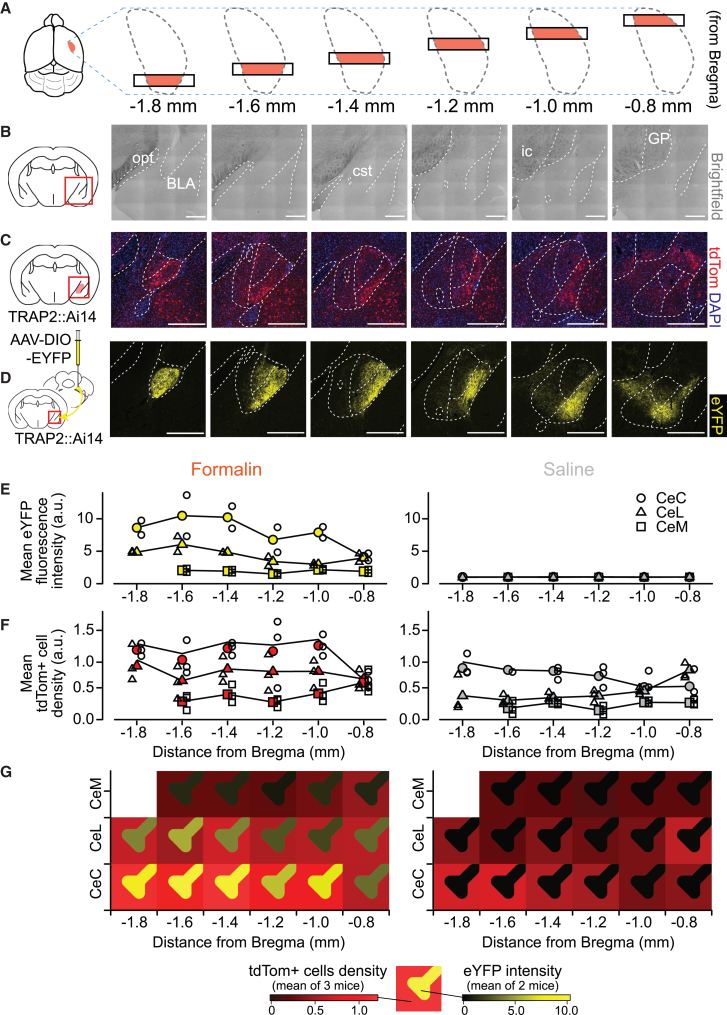
Evaluation of nociTRAPed CeA neuron density and nociTRAPed elPB projection fiber intensity along the rostrocaudal and mediolateral subregional dimensions in the CeA (A–D) Representative brightfield images (B), tdTomato-labeled TRAPed neurons (C), and eYFP-labeled fibers from TRAPed elPB neurons (D) in the CeA at various coronal levels, as illustrated in (A). The brain images were taken from a mouse that received formalin. Anatomical structures are outlined with dashed lines. Scale bars: 200 μm. The coronal level for each mouse was determined based on brightfield observations of anatomical structures, such as the optic tract (opt), commissural stria terminalis (cst), boundaries of the BLA, globus pallidus (GP), internal capsule (ic), and intercalated cell mass in DAPI images. CeA subregion boundary lines were manually drawn according to the corresponding levels in Paxinos and Franklin’s brain atlas (−1.79, −1.55, −1.43, −1.23, −1.07, and −0.83 mm from bregma).^37^ The slices shown in (C) and (D) in Figure 3 represent the same slices shown in Figures 1E1 and 1F1, respectively. (E and F) eYFP fluorescence intensity (E, one section per mouse at each level, two mice per group) and tdTomato+ cell density (F, one section per mouse at each level, three mice per group) in the CeC (circles), CeL (triangles), and CeM (squares) along the rostrocaudal axis. Lines and filled markers represent the mean values. (G) An intuitive summary of postsynaptic neuronal activation and presynaptic projection intensity. The left and right panels summarize the results from the formalin- and saline-injected groups, respectively. Each panel shows a subregion (vertical axis) and rostrocaudal levels (horizontal axis), representing the spatial distribution of the CeA. In each block, the background is colored red based on values from Figure 3F, while synaptic-like shapes are drawn in yellow based on values from Figure 3E. The color scales at the bottom represent tdTomato+ cell density (red-black) and eYFP intensity (yellow-black).

Wilson et al. demonstrated that the distributions of neurons expressing pERK and c-Fos in the CeA vary depending on rostrocaudal location in mice with cuff-induced neuropathic pain in the hind paw.^22^ Therefore, we next compared the expression of tdTomato (representing nociTRAPed soma) and eYFP (representing fibers arising from nociTRAPed elPB neurons) along the rostrocaudal axis (Figure 3). Figure 3A is a rearrangement of coronal slice images along rostrocaudal levels (−0.8 mm to −1.8 mm from the bregma; every 0.2-mm interval). While tdTomato-expressing cells were evenly distributed throughout the rostrocaudal axis (Figure 3F), the intensity of eYFP fluorescence varied unevenly (Figure 3E). The expression of eYFP was significantly higher in the caudal (−1.8 mm to −1.4 mm from bregma) than in the rostral CeA (−1.2 mm to −0.8 mm; Figure S4). Figure 3G graphically summarizes the eYFP fluorescence intensity and the number of tdTomato-expressing neurons in formalin (left panel)- and saline (right panel)-treated animals at distinct rostrocaudal levels. The fibers with strong eYFP signals arising from nociTRAPed elPB neurons were concentrated mainly at levels from −1.4 to −1.8 mm from bregma in the CeC of TRAPed mice with formalin injection (Figure 3G, left-bottom). In contrast, tdTomato signals in CeA neurons could also be found at levels from −1.4 to −1.8 mm from bregma. Moderate fluorescence was also observed at more caudal levels (from −1.2 mm to −1.0 mm) and in the CeL from −1.8 mm to −1.0 mm. Conversely, in saline-treated animals, tdTomato expression was highly limited, and there was almost no eYFP signal throughout the rostrocaudal CeA (Figures 3E, 3F, and 3G). These results suggest that the most robust activation of the elPB-CeC projections is found in the caudal (i.e., posterior) part of the CeC in mice treated with formalin.

### Characteristics and functional roles of FosTRAPed neurons in the CeA

**Figure 4:**
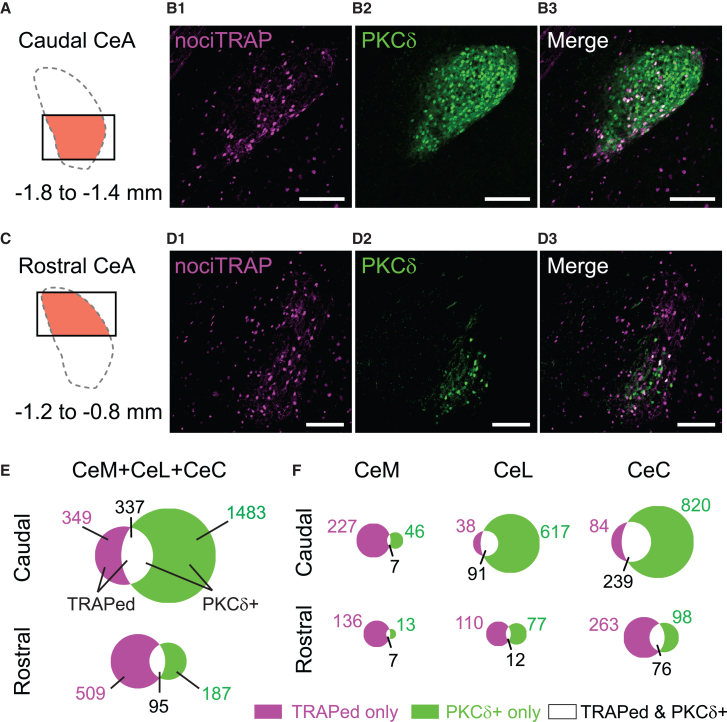
Evaluation of the overlap between PKCδ immunostaining and noci-TRAPed tdTomato expression in the CeA of formalin-injected mice (A–D) Distribution of TRAPed cells (magenta in B1 and D1) and PKCδ+ cells (green in B2 and D2) in coronal sections of the caudal (A and B) and rostral (C and D) CeA of formalin-injected mice. Merged cells are represented in white. The right and top in b and d indicate lateral and dorsal directions, respectively. Approximately −1.8 mm from bregma (B) and −1.2 mm from bregma (D). Scale bars: 200 μm. (E and F) Quantification of cell populations presented as Venn diagrams for the entire CeA (E) and for each subregion of the CeA (F) (Summaries of 19 sections from 2 mice: 9 sections from the caudal CeA, 10 sections from the rostral CeA). Upper row: caudal CeA; bottom row: rostral CeA. In each Venn diagram, magenta, green, and white indicate tdTomato-only, PKCδ-only, and double-positive cells, respectively. Magenta and green numbers represent the total count of tdTomato-only and PKCδ-only cells (i.e., double-positive cells are not included in these counts). Black numbers represent counts of double-positive cells.

The CeA consists of a heterogeneous population of neurons expressing various marker molecules, each playing different roles in pain processing. For example, a subset of CeA neurons expressing δ-type protein kinase C (PKCδ) has been shown to play pro-nociceptive roles.^22^ We examined whether the neurons expressing tdTomato after being fosTRAPed by formalin injection also express PKCδ. Three weeks after FosTRAPing with formalin injection, brain sections of rostral and caudal CeA were immunostained for PKCδ (Figures 4A–4D). As demonstrated earlier (Figures 1F and 3F), the nociTRAPed CeA neurons were found in all subregions of the CeA. However, their overlap with PKCδ-expressing neurons varied depending on the rostrocaudal levels (Figure 4E). We observed more neurons co-expressing PKCδ and tdTomato, particularly in the caudal CeL and CeC, compared to the caudal CeM (Figure 4F). In contrast, we found far fewer double-positive cells in the rostral CeM and CeL (Figure 4F), partly due to the smaller number of PKCδ-expressing neurons in the rostral CeA, consistent with previous reports.^21^^,^^22^ Specifically, in the caudal CeC, a large proportion of FosTRAPed neurons expressed PKCδ (239 of 323 neurons; 74.0%), while in the rostral CeC, only 76 of 339 (22.4%) nociTRAPed neurons expressed PKCδ (Figure 4F). These subregion-dependent differences may reflect the fact that PKCδ-expressing cells are not necessarily the primary receivers of parabrachial nociceptive inputs but rather their roles vary depending on CeA subregions.

We previously demonstrated that formalin injection into the upper lip increases the number of c-Fos-expressing neurons in the right CeA^10^ and induces long-lasting bilateral hind paws sensitization in rats.^23^^,^^28^^,^^38^ Furthermore, we showed that chemogenetic activation of GABAergic neurons in the right CeA alone is sufficient to reduce the mechanical withdrawal threshold in both hind paws, even in the absence of hindlimb injury.^23^^,^^28^^,^^38^ Similarly, chemogenetic activation of PKCδ-expressing neurons in the right CeA also induces bilateral sensitization.^22^ GABAergic neurons constitute a subpopulation of the entire CeA neuronal population, and PKCδ-expressing neurons represent a further subpopulation within the GABAergic CeA neurons. As described previously, the nociTRAPed neurons identified in this study comprise a subpopulation of PKCδ-expressing neurons in the CeA. Based on these findings, we investigated whether selective pharmacological activation of FosTRAPed neurons in the CeA and adjacent regions could reproduce mechanical sensitization in the hind limbs (Figure S5).

AAV vectors encoding hM3Dq were injected into the right CeA 2–3 weeks prior to FosTRAPing (Figures S5A and S5B). Six weeks after subcutaneous formalin injection into the upper lip, we assessed the 50% threshold for paw withdrawal responses (PWT50). Deschloroclozapine (DCZ; 100 μg/kg, i.p.)^39^ was then administered, and PWT50 was re-evaluated 60 min later (Figure S5D). DCZ administration significantly reduced PWT50 in mice expressing hM3Dq in nociTRAPed CeA neurons (Figure S5E), indicating that re-activation of neurons in the right CeA and surrounding areas—previously activated by the initial upper lip formalin injection—can recapitulate the nocifensive behaviors observed during the initial injury, even in the absence of ongoing peripheral inflammation.^24^^,^^40^ Although these data are derived from a preliminary pilot study and remain limited in scope, they nonetheless demonstrate that the nociTRAPed neurons in the CeA and nearby BLA are components of a neuronal ensemble capable of reproducing nociplastic sensitization.

### How do nociTRAPed elPB neurons send nociceptive information to nociTRAPed CeA neurons?

So far, we have demonstrated many nociTRAPed neurons in the elPB and CeA, as well as rich projections from nociTRAPed elPB neurons to the CeA region, particularly to the pCeC. Previously, we demonstrated that a subset of neurons in the lateral parabrachial nucleus forms direct monosynaptic contact with a subset of CeA neurons, particularly those in the CeC, using synapsin- or calca-cre-driven expression of ChR2 and the recording of light-evoked postsynaptic currents in CeC neurons.^8^^,^^41^

The use of fosTRAP2::Ai14 mice in this study allowed us to selectively record synaptic transmission from nociTRAPed elPB neurons to either nociTRAPed or non-nociTRAPed CeA neurons by tdTomato fluorescence-guided patch-clamp recording and light activation of expressed ChR2 in slices. First, to establish this approach, we made patch-clamp recordings and evaluated (1) the fraction of elPB neurons that were TRAPed by formalin injection and (2) the extent to which nociTRAPed and non-TRAPed CeA neurons differ in their electroresponsive properties.

**Figure 5:**
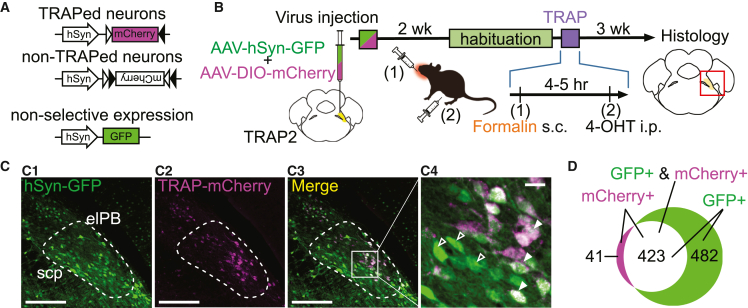
Comparison of neuronal populations between noci-TRAPed expression and non-selective hSyn expression in the elPB (A) Schematic of transfected genes. The middle and bottom constructs were transfected into elPB neurons of TRAP2 mice. After TRAPing, Cre-dependent recombination and mCherry expression occurred in TRAPed neurons, as shown at the top. All neurons infected with the AAV for hSyn-driven expression (bottom) expressed GFP protein. (B) Experimental procedure. AAV-Syn-GFP and AAV-DIO-mCherry were mixed and injected into the elPB simultaneously. Two weeks after the virus injection, mice were habituated for 5 consecutive days. On the day of fosTRAP, mice received orofacial formalin s.c. (1), followed by 4-OHT administration 4–5 h later (2). Brain tissue was collected for histology 3 weeks after fosTRAP. (C) Representative images of the elPB (outlined with dashed lines) in coronal sections (approximately −5.4 mm from bregma). (C1): GFP image; (C2): mCherry image; (C3): merged image of both; (C4): magnified images of the rectangle in (C3). Filled arrowheads represent cells positive for both GFP and mCherry; open arrowheads represent GFP-only cells. Right and top indicate lateral and dorsal sides, respectively. Merged cells are represented in white. Scale bars: 200 μm (C1–C3), 20 μm (C4). (D) Schematic of the populations of GFP-positive and mCherry-positive cells. Magenta represents mCherry-only positive cells, green represents GFP-only positive cells, and white indicates cells positive for both mCherry and GFP. Numbers in the schematic indicate the total number of neurons (four equally spaced brain sections from each of the two mice).

To accomplish the first goal, we prepared a “cocktail” of two different AAV solutions for the expression of eGFP driven by the synapsin promoter (hSyn-eGFP) and mCherry expression in FosTRAPed cells (DIO-mCherry). We injected this cocktail solution into the elPB of TRAP2 mice (Figure 5A) and formalin-TRAPed them two to three weeks later (Figure 5B). Two weeks later, we visualized the fluorescence of eGFP and mCherry in the same slices (Figure 5C).

As expected, many neurons in the elPB expressed eGFP, and mCherry-expressing neurons (i.e., nociTRAPed cells) made up a subpopulation (47%) of these eGFP-expressing neurons (Figure 5D). This result suggests that approximately half of the elPB neurons were TRAPed by formalin treatment.

For the second purpose, we developed a “neighboring pair” recording strategy to compare the electroresponsive properties between nociTRAPed and non-nociTRAPed CeA neurons (Figure S6). These neurons were selectively recorded using mCherry fluorescence as a guiding criterion. In this strategy, we recorded from a pair of neighboring neurons, one with and one without mCherry fluorescence, located less than 30 μm apart. We found that the fractions of “late firing” (Figure S6A) and “regular spiking” (Figure S6B) neurons, classified according to their response to step depolarizing pulse injection (Figure S6C), differed significantly between nociTRAPed and non-nociTRAPed neurons (*p* = 0.039; Fisher’s exact test; odds ratio = 0.31; Figure S6D). This conclusion was further supported by recordings from neurons in neighboring pairs (*p* = 0.023; Fisher’s exact test; odds ratio = 0.11; Figure S6E). These results suggest that c-fos expression following the initial inflammation occurred in a biased population of CeA neurons with distinct membrane excitability. In contrast, we found no significant differences in the resting potential (zero current injection potential) or membrane capacitance between nociTRAPed and non-nociTRAPed CeA cells (Table 1).

**Table 1:** Electrophysiological properties of TRAPed and non-TRAPed CeA neurons

		V_REST_ (mV)		C_m_ (pF)	
		Late firing neurons	Regular spiking neurons	Late firing neurons	Regular spiking neurons
All neurons recorded (M = 9)	TRAPed (*n* = 39; M = 9)	−78.8 ± 3.6 (*n* = 19)	−72.0 ± 7.3 (*n* = 20)∗	56.3 ± 38.9 (*n* = 19)	36.4 ± 24.8 (*n* = 20)∗
	Non-TRAped (*n* = 25; M = 9)	−77.3 ± 4.1 (*n* = 6)	−73.8 ± 5.5 (*n* = 19)∗	47.9 ± 17.6 (*n* = 6)	27.2 ± 22.9 (*n* = 19)∗
Only neurons recorded in neighboring pairs (Figures 7 and S6; *n* = 16; M = 5)	TRAPed	−78.3 ± 2.9 (*n* = 9)	−73.1 ± 6.8 (*n* = 7)∗	44.0 ± 28.2 (*n* = 9)	44.4 ± 28.4 (*n* = 7)∗
	Non-TRAPed	−79.5 ± 0.7 (*n* = 2)	−73.4 ± 5.4 (*n* = 14)∗	42.5 ± 3.9 (*n* = 2)	28.0 ± 23.4 (*n* = 14)∗

∗∗*p* < 0.01; ∗*p* < 0.05; vs. late-firing neurons of the same category, unpaired t test with Benjamini-Hochberg correction for multiple comparisons. The “all neurons recorded” group includes all neurons used for analyses in Figure S6C, except for 26 neurons from 5 mice that received intra-elPB injections of AAV-Syn-ChR2-eYFP, and 18 neurons from 3 mice that received AAV-DIO-ChR2 recorded in pairs, as the same depolarizing protocol used for other neurons in Figure S6 was not performed on these neurons. Additionally, “all neurons recorded” also includes (1) 6 neurons from 2 mice that received AAV-hSyn-ChR2 and 26 neurons from 4 mice that received AAV-DIO-ChR2, recorded in pairs; (2) 3 neurons from AAV-hSyn-ChR2-injected mice and 3 neurons from AAV-DIO-ChR2-injected mice (M = 2 and 2, respectively), recorded not in pairs; and (3) 26 neurons without detectable ChR2-eYFP expression in the CeA and elPB from 3 mice that received AAV-DIO-ChR2 into the elPB. “n” and “M” refer to the number of neurons and mice, respectively, throughout the text.

### NociTRAPed elPB neurons projecting to the CeA form more robust synaptic connections with nociTRAPed CeA neurons

**Figure 6:**
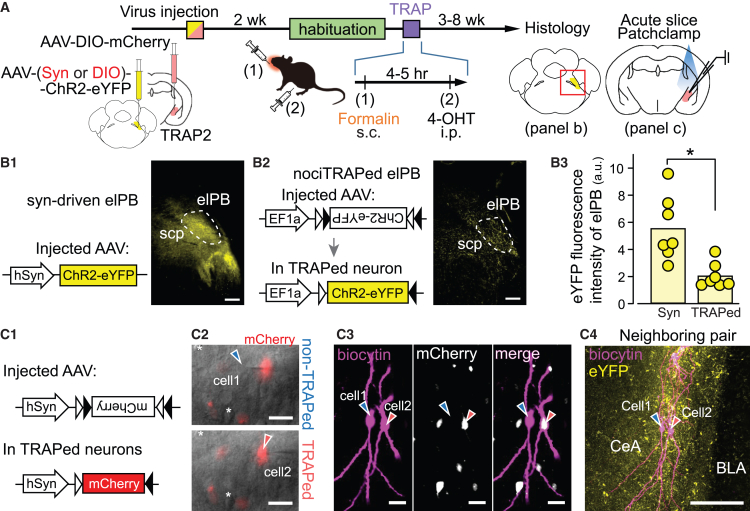
Patch-clamp recording of light-evoked synaptic transmission from nociTRAPed and general elPB neuron populations to nociTRAPed and non-nociTRAPed CeA neurons in neighboring pairs (A) Experimental procedure. Mice received AAV-DIO-mCherry microinjections in the right CeA and AAV-EF1a-DIO-ChR2-eYFP or AAV-hSyn-ChR2-eYFP microinjections in the right elPB simultaneously, followed by formalin treatment. Three to 8 weeks later, acute brain slices containing the right CeA were prepared for patch-clamp recording, and brain blocks containing the pons were fixed for elPB evaluation and histology. (B) elPB histological experiments. (B1 and B2): Left, Schematics of the transfected constructs by AAVs; Right, representative eYFP signal images of brain sections containing the elPB. Dashed lines show the elPB region. (B1): Syn-driven elPB (approximately −5.4 mm from bregma); (B2): TRAPed elPB (approximately −5.4 mm from bregma)) Scale bars: 200 μm. (B3): Comparison of eYFP fluorescence intensity in the elPB (relative to autofluorescence) between both groups. Filled circles indicate individual mice, and bars indicate group averages (∗, *p* = 0.0023; U = 47.0, Mann-Whitney U test. TRAPed elPB: 7 mice; syn-driven elPB: 7 mice). (C) CeA patch-clamp experiments. (C1): schematic of the transfected construct by AAVs. (C2–C4) Representative neighboring cells in the CeA recorded during patch-clamp experiments. Blue-filled and red-filled arrowheads indicate non-TRAPed (cell 1) and TRAPed (cell 2) neurons. (C2): DIC images of the same field of view merged with mCherry image showing a representative example of a “neighboring pair” recording from non-TRAPed (cell 1) and TRAPed (cell 2) neurons. The top and bottom panels show the same field of view with a slight difference in focus; the asterisk indicates the same cell in both images. Scale bars: 20 μm. (C3): representative confocal images of tissue-cleared brain slices containing patched cells. Left: biocytin loaded intracellularly via recording pipettes and visualized with streptavidin Alexa Fluor 647 (magenta); middle: mCherry signal for nociTRAPed neurons (white); right: merged image. Scale bars: 20 μm. (C4): stacked image of tissue-cleared brain slices showing neighboring neurons spreading their dendrites similarly in the brain slice. Yellow: eYFP-ChR2-expressing elPB neuron terminals; magenta: biocytin. Scale bar: 100 μm.

Next, we examined whether nociTRAPed CeA neurons receive synaptic inputs specifically from nociTRAPed elPB neurons. We transfected elPB neurons with two types of AAV vectors for ChR2 expression with distinct expression-driving mechanisms: AAV-hSyn-ChR2-eYFP and AAV-DIO-ChR2-eYFP into the elPB (Figure 6A). The former expresses ChR2 in general elPB neuron populations (Figure 6B1), while the latter limits ChR2 expression to the nociTRAPed elPB neurons (Figure 6B2). The fluorescence intensity of the terminals in the CeA was approximately 2–3 times higher in mice with intra-elPB transfection of AAV-hSyn-ChR2-eYFP than in those with AAV-DIO-ChR2-eYFP (Figure 6B3), consistent with the expression ratio of hSyn-eGFP and DIO-mCherry in nociTRAPed populations (Figure 5D).

Upon visualization of neurons and fluorescence, we recorded postsynaptic excitatory currents (EPSCs) evoked by light stimulation of ChR2-expressing fibers/terminals in multiple neurons. One issue inherent in comparing postsynaptic responses triggered by presynaptic ChR2 activation is the variation in ChR2 levels between different terminals formed at different neurons (as discussed in Sugimura et al. 2016).^8^ To compare postsynaptic responses between TRAPed and non-TRAPed CeA neurons while minimizing this issue, we employed a “neighboring pair recordings” strategy, where one mCherry-expressing CeA neuron and one non-expressing neuron located within 30 μm in the same CeA slice were recorded and compared (e.g., cell1 and cell2 in Figures 6C2–C4), assuming that the axonal length and trajectory from the elPB to CeA would be similar for these neuron pairs.^31^^,^^42^^,^^43^ Indeed, these neuron pairs were within the same “cloud” of eYFP-expressing terminals (Figure 6C4).

First, we compared the distribution of EPSC amplitude between (1) nociTRAPed and non-nociTRAPed CeA neurons and between (2) neurons with hSynapsin-driven (Figure S7A) and nociTRAPed expression of ChR2 (Figure S7B).

When elPB afferents were non-selectively stimulated using hSyn-driven ChR2, the mean leEPSC amplitude of all EPSC events, including failures (mean of all responses μ_ALL_), and that calculated using only successful events (mean of “success” responses μ_RES_), did not differ significantly between nociTRAPed and non-nociTRAPed CeA neurons (Figure S7A3). The ratio of “responder” neurons to all recorded neurons (fraction of responder neurons) also did not differ significantly between TRAPed and non-TRAPed CeA neurons (insert in Figure S7A3).

In contrast, when ChR2 was expressed only in nociTRAPed elPB neurons (Figures S7B1–S7B3), we found a significant difference in μ_ALL_ between TRAPed and non-TRAPed CeA neurons. Additionally, the fraction of responder neurons was significantly smaller in non-nociTRAPed CeA neurons than in nociTRAPed CeA neurons (insert in Figure S7B3). Together, the statistical analysis of EPSC amplitude distribution suggests that nociTRAPed elPB neurons target nociTRAPed CeA neurons more specifically than non-TRAPed neurons.

### NociTRAPed CeA neurons show greater and more robust postsynaptic responses to inputs from nociTRAPed elPB neurons, particularly in the caudal CeC (posterior CeC)

As shown in Figure 3, we found a rostrocaudal bias in the projection pattern of nociTRAPed elPB neurons within the CeA. On the other hand, the distribution of light-evoked EPSC amplitude in nociTRAPed CeA neurons varied widely (Figure S7B3, left). We hypothesized that this large variation in postsynaptic responses in the pooled population was due to the mixture of data from neurons in both rostral and caudal subdivisions of the CeA. Consequently, we made separate comparisons of leEPSC amplitude for neurons in the rostral (recorded in slices from −0.8 mm to −1.2 mm) and caudal (recorded in slices from −1.4 mm to −1.8 mm) regions. Additionally, since we performed neighboring pair recordings of two neurons (one nociTRAPed and one non-nociTRAPed), we plotted the EPSC amplitudes of these pairs to visually analyze the difference between the EPSC amplitudes of nociTRAPed and non-nociTRAPed neurons in the same pair.

**Figure 7:**
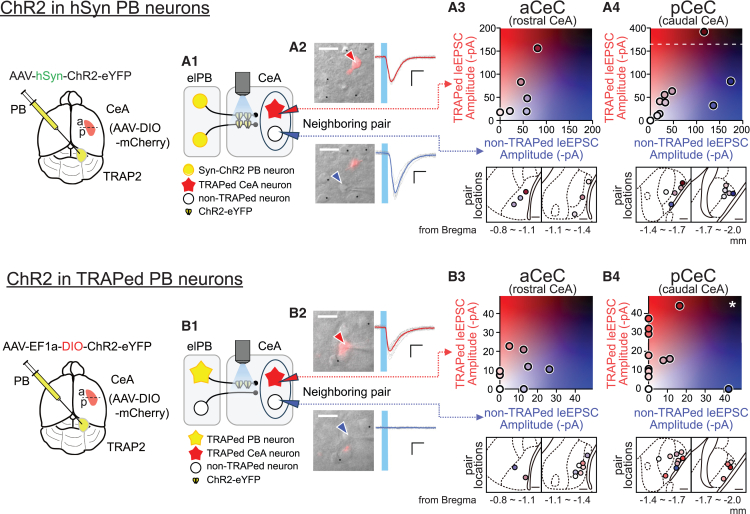
Synaptic transmission between nociTRAPed elPB neurons and nociTRAPed CeA neurons in posterior CeC Optogenetic analysis of elPB-CeC synaptic transmission in mice with hSynapsin-driven (A) and nociTRAPed expression (B) of ChR2-eYFP in elPB neurons. (A1 and B1): schematic illustrations of light-evoked synaptic current recordings from neighboring pairs of nociTRAPed (red star) and non-nociTRAPed (white circle) CeC neurons. Red and blue triangles in the CeA indicate recording pipettes for the nociTRAPed (red) and non-nociTRAPed (blue) CeA neurons, respectively. Expression of ChR2-eYFP was driven by hSynapsin (yellow circles in elPB). (A2 and B2): representative images of whole-cell patch-clamp recordings from mCherry-positive (TRAPed) neurons (top) and neighboring mCherry-negative (non-TRAPed) neurons (bottom). The left panels show DIC images merged with the mCherry signal. Colored arrowheads indicate the recorded neurons (red for TRAPed neurons in the top image, blue for non-TRAPed neurons in the bottom); black asterisks mark the same neurons in both images. Figures above and below show the same view frame. Scale bars: 20 μm. The right panels show representative EPSC traces recorded from the neurons indicated in the left panels. The sky-blue bar indicates the duration of blue-light stimulation. Gray traces represent individual EPSC recordings, while thick-colored traces indicate the average trace of all individual recordings (red for TRAPed neurons, blue for non-TRAPed neurons). Scale bars in (A2) right: vertical, 50 pA, horizontal, 10 ms; scale bars in (B2) right: vertical 20 pA, horizontal 10 ms. (A3 and B3): EPSC amplitude plots of recorded neuron pairs from the anterior CeC (top, 2D color plot). The vertical axis shows EPSC amplitude from TRAPed neurons, while the horizontal axis shows EPSC amplitude from neighboring non-TRAPed neurons. Each colored circle represents the data from a pair of neurons (TRAPed and non-TRAPed), with the color determined by the EPSC amplitude, as shown in the 2D color scale (a3 top, *n* = 6 pairs, *p* = 0.69, Wilcoxon signed-rank test, V = 8; b3 top, *n* = 9 pairs, *p* = 0.80, Wilcoxon signed-rank test, V = 12). Bottom: Spatial distribution of recorded neighboring neuron pairs. Each circle indicates the location of paired neurons located <30 μm apart and is colored according to the color scale of the upper 2D plot. The bottom left and right panels show coronal levels from bregma at −0.8 to −1.1 mm and −1.1 to −1.4 mm, respectively. Scale bars: 100 μm. (A4 and B4): EPSC amplitude plots of recorded neuron pairs from the posterior CeC. The top panels display data in the same manner as in (A3) and (B3). The ∗ in B4 top indicates a significant difference between the leEPSC amplitude of neighboring TRAPed and non-TRAPed neurons (A4 top, *n* = 10 pairs, *p* = 0.64, Wilcoxon signed-rank test, V = 18; B4 top, *n* = 13 pairs, *p* = 0.032, Wilcoxon signed-rank test, V = 12). Bottom: Spatial distribution of recorded neighboring neuron pairs. The bottom left and right panels show coronal levels from bregma at −1.4 to −1.7 mm and −1.7 to −2.0 mm, respectively. Scale bars: 100 μm. The subdivision borders were delineated based on the atlas on representative coronal sections; however, the locations of individual neurons may appear slightly shifted due to minor variations across anatomical planes in (A3), (A4), (B3), and (B4).

The plots in A3, A4, B3, and B4 in Figure 7 depict the *x*-*y* plot of light-evoked EPSC amplitude in nociTRAPed neurons (*y* axis) and non-nociTRAPed neurons (*x* axis) recorded in pairs from the same slices. A3 and B3 show the data from the anterior CeC (aCeC), and A4 and B4 show the results recorded in neurons from the posterior CeC (pCeC). A3 and A4 display data from the hSyn-driven expression of ChR2, while B3 and B4 represent data from the nociTRAPed expression of ChR2. Notably, only in the pCeC with nociTRAPed expression of ChR2 in the elPB neurons did the postsynaptic responses differ drastically between nociTRAPed (*y* axis) and non-nociTRAPed (*x* axis) CeC neurons. This suggests that robust synaptic connections are formed between pain-activated elPB neurons and pain-activated CeC neurons almost exclusively in the posterior CeC. Based on these observations, we propose dubbing this region “the posterior CeC (pCeC)” as the direct entry site for elPB-mediated nociceptive information to the central amygdala.

## Discussion

Using transgenic mice that enabled the selective expression of exogenous proteins such as fluorescent markers (ChR2 and DREADDs) in neurons with a history of c-fos expression within a fixed time window,^26^ we demonstrated that elPB neurons activated during transient inflammation at the upper lip (i.e., “nociTRAPed” elPB neurons) form direct and robust synaptic connections with CeA neurons that were also activated during the same painful event (i.e., nociTRAPed CeA neurons). Specifically, we observed robust synaptic connections between activated elPB neurons and neurons predominantly in the caudal capsular part of the CeA, the pCeC (−1.4 to −1.9 mm from bregma), although the terminals arising from nociTRAPed elPB neurons could be found evenly throughout wide rostrocaudal levels from −1.0 to −1.8 mm from the bregma in the CeA. The elPB-CeA circuit is well recognized as the primary ascending route for nociceptive signals from the spinal dorsal horn, trigeminal spinal nucleus, and trigeminal afferents to the brain.^9^^,^^13^^,^^15^^,^^44^ This view has evolved following recent findings of nociplastic sensitization caused by the activation of this pathway.^23^^,^^24^^,^^28^^,^^40^ Currently, it is considered a pivotal system integrating ascending pain-associated information and descending pain modulation, optimizing sensitivity and behavioral strategies in response to various potentially noxious somatic stimuli.^22^^,^^23^^,^^24^ Our findings provide a foundation for understanding how elPB and CeA neurons connect and co-activate in response to painful events, leading to subsequent nociplastic changes in the pain circuits.

We also found the following: (1) NociTRAPed CeA neurons were distributed throughout the CeC and CeL with a rostrocaudal gradient, coinciding with the distribution of axon terminals from nociTRAPed elPB neurons, particularly in the CeC (Figure 3); (2) elPB neurons exhibited long-distance, widespread projections to brain regions implicated in pain signaling with the densest projection being to the pCeC; (3) a small portion of nociTRAPed CeA neurons expressed PKCδ, particularly in the rostral CeA. In contrast, a larger portion of PKCδ-positive neurons were found in the caudal CeC and CeL (Figure 4); (4) although fewer CeM neurons were nociTRAPed than in the CeC and CeL, a significant number were nociTRAPed (Figure 1), most of which were PKCδ negative (Figure 4); (5) NociTRAPed and non-nociTRAPed CeA neurons exhibited distinct membrane potential responses to step-pulse inputs; and (6) chemogenetic activation of nociTRAPed neurons in the CeA and nearby areas resulted in hindpaw tactile sensitization in freely moving animals (Figure S5).^23^^,^^28^ The mechanisms and functional consequences are discussed in the following text.

### Robust synaptic connection from nociTRAPed elPB neurons to nociTRAPed CeA neurons in the posterior CeC

The most novel and significant finding of this study is the higher rate of synaptic responses in nociTRAPed CeA neurons compared to non-TRAPed CeA neurons in response to light stimulation of nociTRAPed elPB neuron fibers. Notably, this difference was most pronounced in neurons located in the pCeC. This significant distinction in the rate of synaptic connections between nociTRAPed and non-TRAPed CeA neurons in the pCeC was not observed when ChR2 was expressed using the non-specific pan-neuronal promoter synapsin in the elPB. This indicates a stronger and more robust synaptic coupling between elPB and CeA neurons co-activated by nociceptive experiences. Interestingly, while the mean amplitude of successful synaptic transmission (μ_RES_) did not differ between nociTRAPed and non-TRAPed CeC neurons (Figure S7B3), the difference in the rate of synaptic responses (Figure S7B3) suggests that the robust connectivity was based on more substantial anatomical connections between co-activated neurons, rather than differences in synaptic transmission efficiency. In other words, although many neurons in the CeA are activated in response to transient noxious/inflammatory stimuli, the neurons in the pCeC appear to have formed more robust and stable connections with pain-excitable neurons in the elPB.

### How were nociTRAPed CeA neurons without firm connections with nociTRAPed elPB neurons activated by formalin stimulation?

Our preliminary study using retrogradely expressed Cre-recombinase in the nociTRAPed CeA populations, which allowed GFP expression in cells activated during post-formalin stimulation and projecting to excited CeA neurons, indicates that the BLA and subthalamic nucleus exhibited strong GFP expression.^45^ This suggests substantial excitatory influences from non-elPB origins on CeC neuron excitation during sustained painful activation. This result aligns with previous findings of a significantly higher correlation between the number of neurons expressing c-Fos in the CeC and BLA, which was not limited to the caudal CeC.^10^ It is also essential to consider that neuronal excitation, along with the activation of specific receptors such as mGlu receptors, can initiate c-Fos expression in response to synchronized presynaptic activity,^46^ accounting for c-Fos expression in non-pCeC neurons.

### NociTRAPed CeA neurons and PKCδ expression

Among the various molecules expressed in the CeA, PKCδ has been identified as a key regulator of pain-related behaviors.^16^^,^^22^ Our immunostaining in FosTRAP2 mice revealed that approximately 74% of nociTRAPed neurons in the pCeC were PKCδ-positive (Figure 4). However, only about 22.6% of PKCδ-positive neurons in the pCeC were nociTRAPed, suggesting that only a small portion (around one-fifth) of PKCδ-expressing cells in the pCeC were activated by the transient inflammation used in this study. PKCδ has been identified as a marker specific to CeC/CeL, with roles in nociception and fear/threat learning.^47^ This implies that although PKCδ-positive cells are the primary target of nociTRAPed elPB neurons in the pCeC, they represent only a minor subpopulation of all PKCδ neurons. Furthermore, about half of the nociTRAPed neurons recorded in the CeC exhibited late-firing properties (Figure S6). Previous studies showed that approximately 25% of PKCδ neurons in healthy mice have late-firing properties.^21^ Additionally, in formalin-treated rats, late-firing CeA neurons (comprising 36.8% of all recorded CeA neurons) showed significant synaptic potentiation of elPB-CeC transmission 24 h post-formalin.^8^ These findings are consistent with our results, suggesting that transient inflammation activated only a minor subpopulation of PKCδ or late-firing neurons.

However, chemogenetic activation of nociTRAPed CeA neurons was sufficient to induce widespread sensitization, even in the absence of ongoing nociceptor activation at the site of sensitization, indicating nociplastic sensitization. Previous studies have shown that chemogenetic activation of GABAergic neurons in the CeA without tissue damage^23^ or PKCδ-expressing CeA neurons without injury^22^ can induce nociplastic sensitization. Theoretically, PKCδ neurons are a subset of GABAergic neurons in the CeC/CeL,^48^^,^^49^ and nociTRAPed neurons are a subset of PKCδ neurons, as discussed earlier. The number of neurons activated by the DREADD agonist in this study may be the smallest among those showing CeA-driven nociplasticity (pain-resembling behaviors without tissue injury) reported to date, suggesting that nociTRAPed CeA neurons represent the minimal population required for the promotion of nociplastic behaviors.

Moreover, it is worth noting that nociTRAPed neurons outside the CeA might have contributed to the nociplastic behaviors observed following chemogenetic activation. In the present study, hM3Dq-mCherry expression was not strictly limited to the CeA but was also observed in the BLA, particularly in anterior slices (Figure S5C). This finding aligns with our previous report that formalin injection into the upper lip increases the number of c-Fos-expressing neurons not only in the CeA but also in the BLA. Although the expression level was lower in the BLA than in the CeA, the number of c-Fos-expressing neurons was correlated between the CeC and BLA within the same animal,^10^ suggesting a potential causal relationship in their activation. Indeed, subsets of BLA and CeA neurons are synaptically connected,^17^^,^^20^^,^^50^ indicating that these neurons may be co-activated and might form a functional cell assembly involved in regulating pain sensitivity and behavior. It is, therefore, plausible that DCZ-induced activation of hM3Dq excited this assembly, thereby contributing to the observed nociplastic behaviors. A previous study^51^ reported robust expression of eYFP in noci-TRAPed neurons within both the CeA and BLA (Figures S10C and S10G in the study by Corder et al.^51^), following repeated noxious pinprick stimulation to the left hind paw in the fosTRAP mice. In the same study, the authors selectively injected an AAV vector into the BLA to express an inhibitory DREADD specifically in the noci-TRAPed neurons. Chemogenetic inhibition of these BLA neurons selectively suppressed behaviors associated with the affective-motivational dimension of pain in a model of neuropathic pain.^51^ These observations suggest that CeA and BLA neurons—many of which are co-activated under painful conditions but to distinct degrees in distinct pain modalities—may play distinct roles in modulating different dimensions of pain. It is speculated that CeA neurons are more responsive to inflammatory situations, than to the non-inflammatory somatosensory noxious inputs and likely to play a more critical role in regulating reflexive sensitivity via intrinsic brain networks as shown in the present study. It is also likely that this function of the CeA may be essential for the development of nociplastic sensitization in transient inflammatory pain models.^22^^,^^23^^,^^28^

We also found that the capsular part of the CeC is one of the most prominent targets of nociTRAPed elPB neurons in the brain (Figures 3, S2, and S3). Condon et al. demonstrated that repeated chemogenetic activation of Calca-expressing elPB neurons induces long-lasting nociplastic hindlimb sensitization in the absence of tissue injury or neuropathy.^24^ Taken together with our current findings, a plausible scenario is that Calca-expressing elPB neurons are activated by transient inflammation, which in turn excites a subset of CeA neurons—including, but not limited to, those in the pCeC—leading to nociplastic sensitization. The role of CGRP release and its receptor activation in these processes remains to be elucidated in future studies.^19^^,^^23^^,^^24^

Hua et al. described a population of antinociceptive neurons activated by general anesthetics in the bilateral anterior lateral CeA (−0.94 mm to −1.34 mm relative to bregma),^52^ which lies more rostrally than the pCeC defined in the present study. It is unlikely that these neurons had been fosTRAPed as we did not use general anesthetics during the FosTRAP procedure (see STAR Methods).^52^

In the present study, we did not conduct functional manipulations comparing caudal and rostral CeA neurons in behavioral assays. However, a previous report demonstrated the differential contributions of caudal versus rostral *Calcrl*^+^ CeA neurons to anxiety-related behaviors.^53^ In addition, transcriptomic analyses have revealed that gene expression-based neuronal clusters differ substantially between the caudal and rostral CeA.^54^ These findings highlight the growing interest in the distinct functional and molecular characteristics of rostral and caudal CeA subregions, which is further supported by the differences in synaptic organizations clarified in this study. Given the observed co-activation of nociTRAPed elPB neurons, future investigations will aim to elucidate the behavioral roles of nociTRAPed neurons located in the posterior (pCeC) and anterior (aCeC) capsular subdivisions of the CeA.

### Limitations of the study

This study has three main limitations, which should be addressed in future research. First, the current findings are based solely on male mice. It is well established that the phenotypes and mechanisms of various types of pain are sex dependent.^55^ Therefore, determining whether the specific role of the pCeC in promoting nociplasticity is similar in female mice is an important subject for future investigation.

Second, we have presented only data from the right brain hemisphere,^16^^,^^56^^,^^57^ based on previous studies showing right-side-predominant expression of c-Fos in the CeA of mice with transient orofacial inflammation.^10^ Although some fibers from nociTRAPed elPB neurons were observed in the left CeA, comparing nociTRAPed and non-nociTRAPed CeA neurons was difficult, as the c-Fos expression in the left CeA was limited in this model.

Finally, another limitation stems from the use of FosTRAP2 mice, where functional identification of neurons is based on their activation history. Therefore, caution is necessary when interpreting the results, as the observed properties—such as synaptic connectivity—may reflect plastic changes induced by the painful experience rather than the naive organization of the network.

### Functional implications

In human patients, widespread pain and sensitization are hallmarks of primary chronic pain^2^ and nociplastic pain.^3^^,^^58^ The transient orofacial inflammation used in this study causes long-lasting, widespread sensitization.^58^ Thus, the specific involvement of pCeC neurons, as well as the non-specific involvement of elPB and CeC neurons in the initial latent process of widespread sensitization, provides valuable insight into the early priming mechanisms involved in the establishment of nociplastic pain in regions without injury or tissue damage.. The transient orofacial inflammation used in this study causes long-lasting, widespread sensitization.^23^^,^^28^ Thus, the specific involvement of pCeC neurons, as well as the non-specific involvement of elPB and CeC neurons in the initial latent process of widespread sensitization, provides valuable insight into the early priming mechanisms involved in the establishment of nociplastic pain in regions without injury or tissue damage.

In healthy humans, the elPB-CeA pathway's activity critically represents stimuli's aversiveness.^13^ Functional connectivity between the medial prefrontal cortex, nucleus accumbens, and amygdala is increased in patients with a three-year history of spontaneous back pain.^4^ However, these studies describe already-established chronic pain states, and the processes leading to such states remain poorly understood. The present results offer a novel framework for understanding the bottom-up nociplastic processes^58^ that occur after initial inflammation or nociception, potentially leading to widespread, established pain in future human studies using high spatial and temporal resolution. that occur after initial inflammation or nociception, potentially leading to widespread, established pain in future human studies using high spatial and temporal resolution.

## Resource availability

### Lead contact

Further information and requests for resources and reagents should be directed to and will be fulfilled by the lead contact, Fusao Kato (fusao@jikei.ac.jp).

### Materials availability

No unique regents and materials were generated in this study.

### Data and code availability

• Data reported in this paper are available from the lead contact upon request, subject to their approval.
• All original code is available from the lead contact upon request, subject to their approval.
• Any additional information required to reanalyze the data reported in this paper is available from the lead contact upon request, subject to their approval.

## Acknowledgments

This work was supported by Grant-in-Aid for Exploratory Research from the Ministry of Education, Culture, Sports, Science and Technology to F.K. (no. 23650208), MEXT-Supported Program for the Strategic Research Foundation at Private Universities (S1311009) to F.K., AMED under grant no. 21ek0610026h0001 to F.K., and by JSPS KAKENHI Grants to F.K. (18H02722, 21H02816, 25293136), Grant-in-Aid for Scientific Research (B) to F.K. JSPS Grant-in-Aid for Scientific Research (B) grant no. 21H02816 to F.K., Grant-in-Aid for Scientific Research (C) to Y.T (20K09207, 23K08391), The Nakatomi Foundation and Takeda Science Foundation to Y.T., and The Jikei University Research Fund for graduate students to T.O. Dr. Ayako M Watabe (Jikei University School of Medicine) provided the Ai14 mice.

## Author contributions

Conceptualization, T.O., Y.T., and F.K.; methodology, T.O., S.U., Y.K.S., Y.T., and F.K.; software, T.O. and F.K.; formal analysis, T.O., Y.T., and F.K.; investigation, T.O., S.U., and Y.T.; writing – original draft, T.O.; writing – review and editing, Y.T. and F.K.; visualization, T.O. and N.S.; funding acquisition, T.O., Y.T., and F.K.; resources, Y.T., M.T., and F.K.; supervision, M.T. and F.K.

## Declaration of interests

The authors declare no competing interests.

## Declaration of generative AI and AI-assisted technologies in the writing process

While preparing this manuscript, the authors utilized Grammarly and ChatGPT only for the purpose of enhancing the quality, readability and flow of the English text written initially by the authors in English. For the graphical abstract, Biorender was used. The authors have reviewed and edited the content and take full responsibility for the final version of the publication.

## STAR★Methods

### Key resources table

**Table:** 

REAGENT or RESOURCE	SOURCE	IDENTIFIER
**Antibodies**		

Rabbit monoclonal anti-PKCδ	Abcam	Cat#ab182126; RRID: AB_2892154
Goat Alexa Fluor 488-conjugated anti-rabbit IgG	Thermo Fisher Inc.	Cat#A11008; RRID: AB_143165

**Bacterial and virus strains**		

pAAV-hSyn-hChR2(H134R)-EYFP	Karl Deisseroth	Addgene AAV5; #26973-AAV5
pAAV-EF1a-double floxed-hChR2(H134R)-EYFP-WPRE-HGHpA	Karl Deisseroth	Addgene AAV5; #20298-AAV5
pAAV-hSyn-EGFP	Bryan Roth	Addgene AAV5; #50465-AAV5
pAAV-hSyn-DIO-mCherry	Bryan Roth	Addgene AAV5; #50459-AAV5
pAAV-hSyn-DIO-hM3D(Gq)-mCherry	Bryan Roth	Addgene AAV5; #44361-AAV5

**Chemicals, peptides, and recombinant proteins**		

Medetomidine hydrochloride	Zenoaq	CAS:86347-15-1
Midazolam	Astellas	CAS:59467-70-8
Butorphanol tartrate	Meiji Seika Pharma	CAS:58786-99-5
Ropivacaine	Aspen	CAS:84057-95-4
Atipamezole hydrochloride	Zenoaq	CAS:104075-48-1
4-Hydroxy tamoxifen	Sigma-Aldrich	Cat#H6278; CAS:68392-35-8
37% formaldehyde solution	Nacalai Tesque	CAS:50-0-0
Isoflurane	Viatris	CAS:26675-46-7
DAPI solution	DOJINDO	Cat#D523; CAS:28718-90-3
Biocytin	Nacalai Tesque	Cat#04856-32; CAS:576-19-2
Streptavidin Alexa Fluor™ 647 conjugate	Thermo Fisher Scientific Inc	Cat#S21374; RRID: AB_2336066
Tissue-Clearing Reagent CUBIC-L	Tokyo Chemical Industry Co.	Cat#T3740
Tissue-Clearing Reagent CUBIC-R+(M)	Tokyo Chemical Industry Co.	Cat#T3741
Deschloroclozapine (DCZ)	ChemScene LLC	Cat#CS-M3647; CAS:1977-07-7

**Experimental models: Organisms/strains**		

Mouse: B6.129(Cg)-*Fos*^*tm1.1(cre/ERT2)Luo*^/J	The Jackson Laboratory	RRID:IMSR_JAX:021882
Mouse: STOCK *Fos*^*tm2.1(icre/ERT2)Luo*^/J	The Jackson Laboratory	RRID:IMSR_JAX:030323
Mouse: B6.Cg-*Gt(ROSA)26Sor*^*tm14(CAG-tdTomato)Hze*^/J	The Jackson Laboratory	RRID:IMSR_JAX:007914

**Oligonucleotides**		

Primers: Genomic PCR, see The Jackson Laboratory website	Invitrogen	N/A

**Software and algorithms**		

Igor Pro 9 (with home-made macros)	WaveMetrics	https://www.wavemetrics.com/software/igor-pro-9
SciPy Python module	Virtanen et al.^59^	https://scipy.org/
EZR	Kanda^60^	https://www.jichi.ac.jp/usr/hema/EZR/statmed.html
ImageJ (Fiji)	Schindelin et al.^61^	https://imagej.nih.gov/ij/
LabChart 8	ADInstruments	https://www.adinstruments.com/products/labchart

### Experimental model and study participant details

#### Animals

TRAP1, TRAP2, and Ai14 mice were purchased from The Jackson Laboratory (#021882, #030323, #7914, Bar Harbor, ME, USA). TRAP1 mice were crossed with C57BL/6J mice and maintained as heterozygotes. The second generation of adult male TRAP1 mice (12–15 weeks old) was used exclusively in the experiments presented in Figures S1A–S1E, which were designed to assess the kinetics of Cre-mediated gene expression following formalin injection and 4-OHT administration. TRAP1 mice were not used in experiments where the expression levels of the gene products matter. TRAP2 mice homozygous for the TRAP2 allele (TRAP2/TRAP2) were bred with each other to maintain a homozygous line. Adult male TRAP2 mice (8–15 weeks old) were used for all other experiments. Ai14 mice were maintained and backcrossed to C57BL/6 for over ten generations by Dr. Ayako M. Watabe. TRAP2::Ai14 mice were produced by crossing TRAP2 and Ai14 mice and were finally maintained as homozygotes for both genes. Adult male TRAP2::Ai14 mice (8–12 weeks old) were used for experiments (Figure 1A). Mice were group-housed with no more than four per cage under a 12:12 h light/dark cycle. Food and water were provided *ad libitum*. Mice were individually housed from three days before TRAP to one day after TRAP and then returned to group housing with the same population. Sibling mice were randomly separated into formalin- and saline-injected groups for histological experiments (Figures 1, 2, and S1).

#### Statement of ethics

The manipulation of the animals was approved by the Institutional Animal Care and Use Committee of Jikei University (No. 2018-013, 2021-044, 2024-018) and conformed to the Guidelines for Proper Conduct of Animal Experiments of the Science Council of Japan (2006) and the guidelines of the International Association for the Study of Pain^62^

### Method details

#### Virus vector injection

Mice were initially anesthetized with 5% isoflurane in 100% oxygen. After the righting reflex disappeared, mice were administered a mixture of medetomidine hydrochloride (0.3 mg/kg, i.p.; Zenoaq, Orion Corporation, Espoo, Finland), midazolam (4.0 mg/kg, i.p.; Astellas, Tokyo, Japan), and butorphanol tartrate (5 mg/kg, i.p.; Meiji Seika Pharma, Tokyo, Japan) intraperitoneally.^63^ Mice were mounted in a stereotaxic frame (Narishige, Tokyo, Japan). Under topical infiltration of ropivacaine (0.75%, 0.3 mL; Anapeine, Aspen JAPAN), an incision was made to expose the skull surface. A hole was drilled, taking care not to severely injure the dura. Virus suspensions containing an AAV (serotype 5) encoding one of the following five constructs were injected with a 10-μL Hamilton microsyringe with a 33-gauge needle (1701RN Neuros Syringe, Hamilton, NV, USA) into the right CeA (500 nL) or right elPB (300 nL): 1) AAV-hSyn-hChR2(H134R)-EYFP (#26973-AAV5, Addgene, MA, USA), 2) AAV-EF1a-double floxed-hChR2(H134R)-EYFP (#20298-AAV5, Addgene, MA, USA), 3) AAV-hSyn-EGFP (#50465-AAV5, Addgene, MA, USA), 4) AAV-hSyn-DIO-mCherry (#50459-AAV5, Addgene, MA, USA), 5) AAV-hSyn-DIO-hM3D(Gq)-mCherry (#44361-AAV5, Addgene, MA, USA). Before microinjection, viral vectors were diluted with PBS or carbogen-saturated ACSF and adjusted to 3-5 × 10^12^ GC/mL. The stereotaxic coordinates for injection into the right CeA were 1.3 mm posterior and 2.95 mm lateral from the bregma and 4.85 mm ventral to the skull surface. For injection into the right elPB, the coordinates were 3.5 mm posterior and 1.5 mm lateral from the bregma, and 4.4 mm ventral to the skull surface with a 20-degree rostral angle. The solution was injected at 100 nL/min using a microsyringe pump (Ultra-MicroPump II with SYS-Micro4 Controller, UMP2, UMC4, World Precision Instruments, FL). The injection syringe remained in place for 5 min after the injection to allow virus diffusion before withdrawal. After suturing the incision, mice were administered atipamezole hydrochloride (3 mg/kg, i.p.) to antagonize the effects of medetomidine and warmed to aid recovery. Once fully awake, mice were housed individually for 3-5 days to allow for wound healing and then returned to group housing. By the time of the TRAP experiment (3–4 weeks after AAV injection), all mice’s skull wounds had healed.

#### Preparation of 4-OHT solution

4-Hydroxy tamoxifen (4-OHT) (H6278, Sigma-Aldrich) was diluted (100 mg/mL) in dimethyl sulfoxide (DMSO) (D8418, Sigma-Aldrich) and stored at −30°C. Immediately before administration, the stock solution was thawed at room temperature and mixed well with polyoxyethylene (20) sorbitan monooleate (Tween-80) (161–21621, Fujifilm Wako Chemicals, Osaka, Japan). The solution was then mixed with saline to make a clear solution. The final 4-OHT solution (5 mg/mL 4-OHT, 5% DMSO, 5% Tween-80) was administered to animals within 10 min.

#### FosTRAP in post-formalin late-onset sensitization model

Sibling mice were randomly assigned to the “formalin” or “saline” groups to minimize genetic variability. Before the orofacial injection of formalin or saline and intraperitoneal administration of 4-OHT, the mice underwent daily handling for one week and were habituated to the posture required for these injections once or twice daily for five days (Figures 1C and 1D). On the day of “TRAP,” each mouse was gently taken from the home cage, held, and injected with 10–20 μL of 5% formalin (37% formaldehyde solution (Nacalai, Kyoto, Japan) diluted with saline (0.9% NaCl)) into the right upper lip using a microsyringe with a 30-gauge needle (Becton Dickinson and Company, Fukushima, Japan)^64^ in the “Formalin” group. Mice in the “Saline” group received an equal volume of saline into the right upper lip. Immediately after the formalin or saline injection, the mice were returned to their home cages. 4-OHT was administered 4–5 h after the formalin or saline injection, except for the experiment shown in Figure S1B and S1C, in which, effects of 4-OHT injection 2 h after formalin was compared with that at 5 h. All formalin or saline injections and 4-OHT administrations were performed without anesthetics, as it has been reported that neurons in the central amygdala express c-fos under general anesthesia.^52^

#### Preparation of transverse brain slices for electrophysiological recording

From 3 to 8 weeks after TRAP, coronal brain slices containing the amygdala were prepared as previously described.^8^ Briefly, the mice were anesthetized with 5% isoflurane (100% O_2_) and transcardially perfused with ice-cold cutting solution containing (in mM): 2.5 KCl, 0.5 CaCl_2_, 10 MgSO_4_, 1.25 NaH_2_PO_4_, 2 thiourea, 3 sodium pyruvate, 92 N-methyl-D-glucamine, 20 HEPES, 12 N-acetyl-L-cysteine, 25 D-glucose, 5 L-ascorbic acid, and 30 NaHCO_3_ (pH ∼7.3, equilibrated with 95% O_2_ + 5% CO_2_; osmolality ∼280 mOsm/kg). After 2–3 min of perfusion, the brain was dissected and bisected at the midline in an ice-cold cutting solution. Each hemisphere was secured on a cutting stage in a vibrating blade slicer (Neo Linear Slicer MT, Dosaka EM, Kyoto, Japan), with the rostral end upwards. Coronal slices (300 μm thick) containing the amygdala were cut in the same ice-cold cutting solution. The slices taken from the right hemisphere were stored in a holding chamber at 34.5°C in the cutting solution for 10–15 min. Subsequently, they were transferred to another holding chamber containing standard artificial cerebrospinal fluid (ACSF) (in mM): 125 NaCl, 3 KCl, 2 CaCl_2_, 1.3 MgCl_2_, 1.25 NaH_2_PO_4_, 10 D-glucose, 0.4 L-ascorbic acid, and 25 NaHCO_3_ (pH 7.4, equilibrated with 95% O_2_ + 5% CO_2_; osmolarity ∼310 mOsm/kg) and kept at room temperature (20°C–25°C) until electrophysiological recordings. Brainstem tissue was collected and stored in 4% PFA for further elPB histology.

#### Patch-clamp recordings from CeC/L neurons

Each brain slice was transferred to a recording chamber (∼0.4 mL volume) and secured with nylon grids on a platinum frame. The slices were continuously superfused at a rate of 1–2 mL/min with standard ACSF. Capsular and lateral parts of the central amygdala (CeC/L) neurons were visually identified using an upright microscope (BX-51WI, Olympus, Tokyo, Japan) with oblique illumination. Images were captured using a charge-coupled device camera (IR-1000; DAGE-MTI) and stored digitally. Whole-cell transmembrane currents were recorded from right CeC/L neurons. Patch-clamp electrodes were made from borosilicate glass pipettes (1B120F-4; World Precision Instruments, FL, USA) with tip resistances of 4–8 MΩ. The internal solution contained (in mM): 120 potassium gluconate, 6 NaCl, 1 CaCl_2_, 2 MgCl_2_, 2 ATP-Mg, 0.5 GTP-Na, 12 phosphocreatine-Na_2_, 5 EGTA, 5 QX-314, and 10 HEPES hemisodium (pH 7.3 adjusted with KOH; osmolarity ∼300 mOsm/kg). Biocytin (0.2%) was added for post-experimental visualization of neurons in a part of recordings. For light-evoked excitatory post-synaptic currents (leEPSCs), a 473 nm LED was illuminated through a 40× water immersion objective lens for 5 ms using the Lumencor Spectra 6 Light Engine (Lumencor, OR, USA), controlled by Master 9 (A.M.P.I., Jerusalem, Israel). leEPSCs were recorded at a holding potential of −60 mV using voltage-clamp recording. leEPSC amplitudes were calculated and averaged over 8–10 sweeps using custom-built functions in Igor Software (WaveMetrics, OR, USA). Neurons with calculated amplitudes below 5 pA or no clear response were considered non-responders. Membrane voltage recordings under current-clamp conditions were used to assess firing patterns. Voltage recordings at around −60 mV were obtained during 2-s initial hyperpolarizations (below −80 mV), followed by 500 ms of 20 pA step currents, up to 2× the rheobase. Latency measurements at 2× rheobase sweeps were performed manually using Igor Pro 8 and 9 software (WaveMetrics, OR, USA). Data were recorded with a Multiclamp 700B amplifier (Molecular Devices, CA, USA), low-pass filtered at 2 kHz, sampled at 10 kHz using a PowerLab interface (AD Instruments, Dunedin, New Zealand), and stored using Lab Chart Software (AD Instruments, Dunedin, New Zealand). All recordings were conducted at room temperature (20°C–25°C). All compounds, except those noted above, were purchased from Sigma (MO, USA) or Nacalai Tesque (Kyoto, Japan).

#### Histology

Mice were transcardially perfused with ice-cold phosphate-buffered saline (PBS), followed by 4% paraformaldehyde (PFA) in 0.1 M phosphate buffer (pH 7.4), under isoflurane anesthesia (5% in 100% O_2_). After post-fixation in 4% PFA at 4°C overnight, the brains were cryoprotected in 20% sucrose in PBS for 24–48 h at 4°C. The brain blocks were embedded in OCT Compound (Sakura Finetek, Tokyo, Japan) and stored at −80°C. Coronal sections (25 μm thick) containing the CeA or elPB were prepared using a cryostat (CM1850, Leica Biosystems, Tokyo, Japan). Every four or eight sections were collected in 0.01 M PBS with 0.05–0.1% sodium azide. Sections were mounted on glass slides, washed with 0.02 M PB, and air-dried. Sections were then immersed in a 1:500-1:1000 DAPI solution for 15 min, followed by three PB washes. After air drying, sections were embedded in Aqua Poly/Mount anti-fading medium (Polysciences, PA, USA) with coverslips and stored at 4°C until imaging.

#### Neuron staining and tissue clearing

After patch-clamp recording, acute brain slices containing biocytin-labeled cells were fixed in 4% PFA in 0.1 M PBS overnight at 4°C. The slices were washed with 0.3% Triton X-100 in PBS three times for 10 min each and then incubated with Streptavidin Alexa Fluor™ 647 conjugate (Thermo Fisher Scientific Inc., USA) (1:1000 dilution) in 0.3% Triton X-100 PBS at 4°C overnight.

The fixed brain slices were cleared using the CUBIC L/R+ reagent (TCI, Tokyo Chemical Industry Co., Ltd., Japan).^65^ Slices were washed with PBS three times for one hour and then incubated with CUBIC L reagent, shaking gently at 37°C in the dark overnight. After washing with PBS for one hour three times, slices were incubated in CUBIC R+ reagent, shaking gently at room temperature in the dark overnight.

Images were acquired using confocal microscopy (FV1200, Olympus, Tokyo, Japan) at every 2-micron depth (10× objective zoom lens, 1024×1024 pixels, Olympus, Tokyo, Japan). The images shown in Figure 6C4 were Z-stacked using the max intensity method in ImageJ Fiji software.^61^^,^^66^

#### Cell count and quantification

Tissue images were visualized using an upright fluorescent microscope (BX63, Olympus, Tokyo, Japan). Grayscale (8-bit) images were captured with a c-MOS camera (4-10× objective zoom lens, 1360×1024 pixels, DP80, Olympus, Tokyo, Japan) and saved as 8-bit TIFF files. The captured bright-field and DAPI images were used to identify anatomical structures such as the optic tract, commissural stria terminalis (cst), boundaries of the BLA, the globus pallidus, and the intercalated cell mass. The rostrocaudal plane was determined by comparing these landmark structures with a brain atlas.^37^ Based on these identifications, boundary lines for sub-regions of the CeA were manually drawn on each section.

Color images (RGB) were constructed using magenta and green pseudocolor space, in which overlapping regions appear white. In images depicting eYFP-expressing neurons and fibers in relation to tdTomato-expressing nociTRAPed neurons, yellow and red pseudocolors were used to maintain consistency with the schematic drawings (e.g., Figures 2 and 3). For Figure 2C1 and 2C2, which show eYFP (yellow)- and tdTomato (red)-signals, an additional set of images was generated using green and magenta pseudocolor space to better separate overlapping signals (Fig. S8). All pseudocolor conversions were performed using ImageJ.

TRAPed cell counts were performed as described previously,^10^^,^^67^ in a semi-blinded manner using ImageJ. Briefly, 8-bit fluorescent images were processed using a median filter to reduce noise (“Despeckle”) and converted to binary images by thresholding with the “Triangle” algorithm, with manual corrections applied only when necessary. Images from both experimental groups were pooled and randomly displayed without revealing their identity. Particles larger than 50 μm^2^ and with a circularity greater than 0.2 were counted using the “analyze particle” function in ImageJ.

#### Immunostaining

TRAP2::Ai14 mice were TRAPed with orofacial formalin injections as described in the “FosTRAP in post-formalin late-onset sensitization model” section. Two weeks after the procedure, the mice were transcardially perfused with PBS followed by 4% PFA. Brains were collected and post-fixed in 4% PFA overnight, then cryoprotected with 20% and 30% sucrose in PBS, sequentially. The brains were sectioned at 25 μm thickness using a cryostat (CM1850, Leica Biosystems, Tokyo, Japan). Sections were collected every 100 μm in PBS, washed with 0.3% Triton X-100 PBS three times, and blocked with a solution containing 1% normal goat serum, 0.3% Triton X-100, and 1% bovine serum albumin in PBS for 1 h at room temperature. The sections were then incubated overnight at 4°C with a rabbit anti-PKCδ antibody (1:1000 dilution, ab182126, Abcam, England). After primary antibody incubation, the sections were washed three times with 0.3% Triton X-100 PBS and incubated with Alexa Fluor 488-conjugated goat anti-rabbit IgG (1:1000 dilution, A11008, Thermo Fisher Inc., USA) for 1 h at room temperature. Following secondary antibody incubation, the sections were washed three times with PBS and mounted on glass slides for microscopy.

#### Evaluation of mechanical sensitization using von Frey filaments

Six weeks after intra-CeA injection of AAV-hSyn-DIO-Gq-mCherry or AAV-hSyn-DIO-mCherry virus, mechanical sensitivity at the hind paws was evaluated using von Frey filaments.^23^^,^^28^^,^^41^ Mice were habituated on the mesh floor before testing. On the test day, mice were administered deschloroclozapine (DCZ) (100 μg/kg, i.p.). Thirty to 120 min after DCZ administration, mice were placed in individual chambers with a mesh floor. The von Frey test was performed to estimate a 50%-threshold for paw withdrawal responses (PWT50) at both hind paws using calibrated von Frey filaments (0.008–2 g; North Coast Medical Inc., Gilroy, CA, USA) and up-and-down methods.^68^ Experimenters were blinded to the treatment history of each mouse.

### Quantification and statistical analysis

Statistical analysis was performed using the SciPy Python module^59^ and EZR software.^60^ A *p*-value of <0.05 was considered statistically significant. All statistical tests were two-tailed. Comparisons between two groups were conducted using unpaired t-tests for direct measurements, the Mann-Whitney U-test for values with non-linear, monotonically increasing conversions (e.g., histological evaluations), or Wilcoxon’s sign test for paired comparisons. Comparisons between multiple groups, or groups with two factors, were performed using the Kruskal-Wallis and two-way ANOVA tests, respectively, with *p*-values adjusted using Benjamini & Hochberg procedures. PWT50 values were log-transformed to ensure normal distribution. Comparisons of fractions were made using Fisher’s exact probability test (expected frequency, *n* < 10).
