# Supplementary material for: The posterior capsular central amygdala showing synaptic coactivation with nociplastic pain-associated parabrachial neurons in mice
Source: iScience. 2025 Jun 25;28(8):113001. doi: 10.1016/j.isci.2025.113001 (PMC12335968; doi:10.1016/j.isci.2025.113001)
Supplement: Document S1. Figures S1–S8 [file mmc1.pdf]

iScience, Volume *28*

Supplemental information

The posterior capsular central amygdala showing synaptic coactivation with nociplastic pain-associated parabrachial neurons in mice

Takao Okuda, Sawako Uchiyama, Naoko Sato, Yae K. Sugimura, Yukari Takahashi, Makoto Tsuda, and Fusao Kato

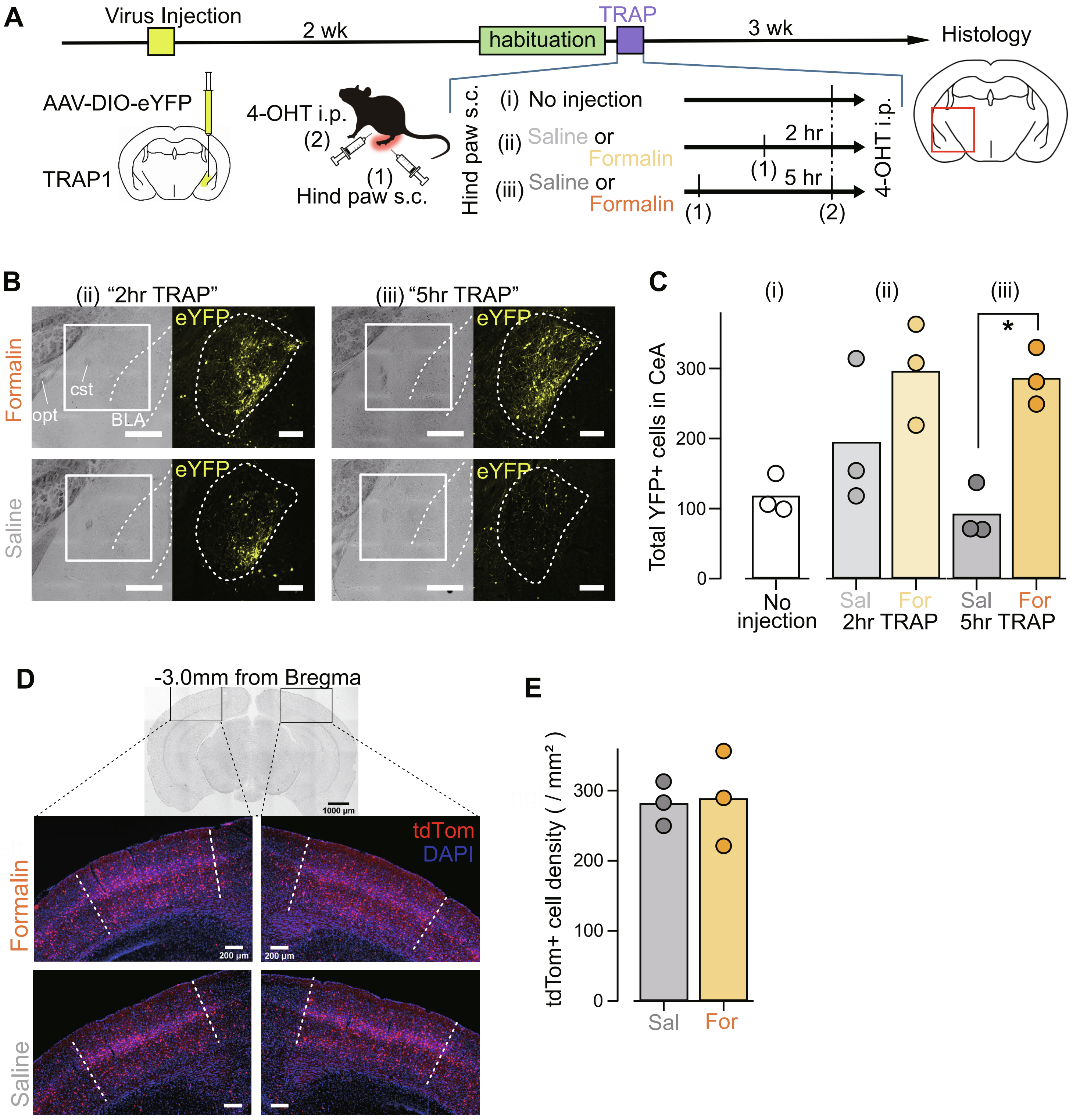

A
Virus Injection
2 wk
habituation
TRAP
3 wk
Histology
AAV-DIO-eYFP
TRAP1
4-OHT i.p.
(2)
(1)
Hind paw s.c.
Hind paw s.c.
(i) No injection
(ii) Saline or Formalin
(iii) Saline or Formalin
2 hr
5 hr
(1)
(2)
4-OHT i.p.
B
(ii) "2hr TRAP"
(iii) "5hr TRAP"
Formalin
Saline
eYFP
cst
opt
BLA
C
(i)
(ii)
(iii)
Total YFP+ cells in CeA
300
200
100
0
No injection
Sal
For
2hr TRAP
Sal
For
5hr TRAP
D
-3.0mm from Bregma
1000 µm
tdTom
DAPI
Formalin
Saline
200 µm
200 µm
E
tdTom+ cell density (/ mm²)
300
200
100
0
Sal
For

Figure S1

Figure S1. Influence of timing of 4-OHT-induced fosTRAPing after formalin injection on Cre-dependent eYFP expression and assessment of nociTRAPed neurons in the primary visual cortex, Related to Figure 1.

A: Experimental timeline for evaluating the effect of the interval between formalin injection and 4-hydroxytamoxifen (4-OHT) administration on Cre-dependent eYFP expression in TRAP1 mice. All animals received an injection of AAV-DIO-eYFP into the right CeA two weeks prior to habituation and subsequent TRAPing (1A). Group (i), no formalin injection, served as a control and received 4-OHT without prior formalin treatment, remaining undisturbed in the home cage until the day of TRAPing. Group (ii): “2-hour interval”, received 4-OHT 2 hours after intraplantar injection of formalin or saline into the left hindpaw. Group (iii): “5-hour interval”, received 4-OHT 5 hours after the same treatment. Brains were collected for histological analysis three weeks after the TRAPing procedure.

B: Representative images (approximately –1.4 mm from Bregma) of the CeA from formalin-injected (upper panels) and saline-injected (lower panels) mice, TRAPed at 2 hours (left panels; experiment ii) or 5 hours (right panels; experiment iii) after injection. In each panel set, the left image shows a bright-field view highlighting the anatomical structures around the CeA, while the right image shows eYFP expression in the area outlined by a rectangle in the corresponding left image. White dotted lines delineate the CeA region. opt, optic tract; cst, commissural stria terminalis; BLA, basolateral amygdala. Scale bars: 500 μm (left panels), 200 μm (right panels).

C: Total TRAPed neuron counts in the right CeA for each mouse in each group (12 sections/mouse, 3 mice/group). Treatment factors (formalin vs. saline) and time factors (2-hour TRAP vs. 5-hour TRAP) were assessed using a two-way ANOVA (Treatment factor: $p = 0.0062$, $F(1,8) = 13.5547$; Time factor: $p = 0.198$, $F(1,8) = 1.97$; Treatment*Time: $p = 0.281$, $F(1,8) = 1.3345$). Statistical coparison with * indicates a significant difference between the saline-treated and formalin-treated "5-hour TRAP" groups ($p = 0.0039$, adjusted with the Benjamini-Hochberg method for multiple comparisons).

D: Representative images of the primary visual cortex, V1 area in TRAP2::Ai14 mice TRAPed at 5 hours after formalin or saline injection. The top image shows a bright-field

image of the V1-containing brain section at the coronal level around -3.0 mm from Bregma. For cell density normalization (Figs. 1, 2), the tdTomato-positive cell density on both sides of the V1 was calculated. The black scale bar indicates 1000 μm. The middle and bottom rows show representative images of the tdTomato-expressing V1 area in formalin-TRAPed and saline-TRAPed mice, respectively. The fluorescence images in the middle row show the V1 area outlined by a rectangle in the top panels. The white dashed line indicates the border of the V1 region, and the white scale bars indicate 200 μm. The bottom row shows images from saline-TRAPed mice, at the same scale as the images in the middle row.

E: Summary of tdTomato-positive cell density (/mm^2). Each dot represents the average density on both sides. The bars show the mean of three mice in each group (12 sections/mouse, 3 mice/group). unpaired t-test: $t(4) = 0.168$, $p = 0.88$.

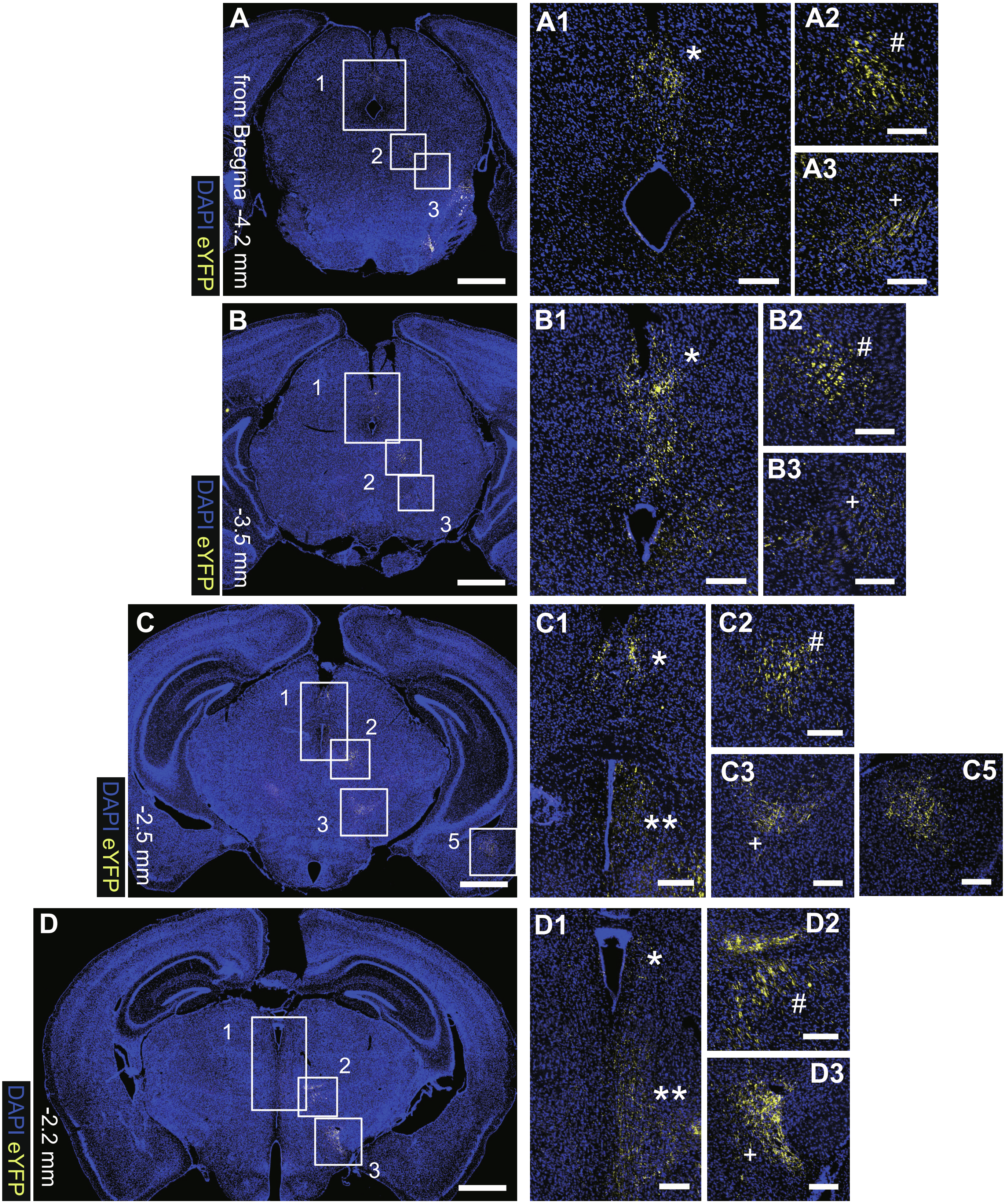
A
A1
A2
A3
from Bregma -4.2 mm
DAPI eYFP
B
B1
B2
B3
-3.5 mm
DAPI eYFP
C
C1
C2
C3
C5
-2.5 mm
DAPI eYFP
D
D1
D2
D3
-2.2 mm
DAPI eYFP

Figure S2

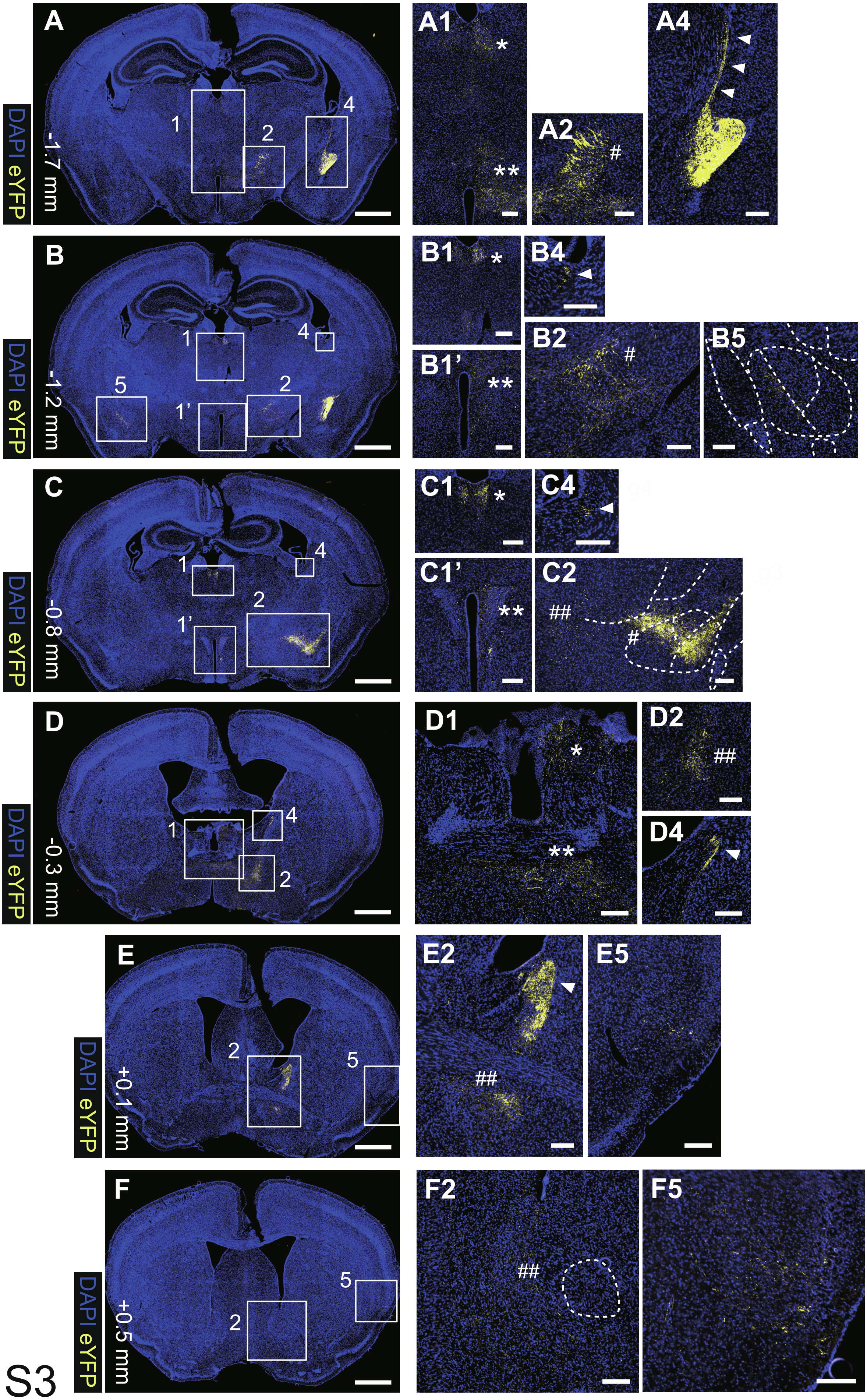

A
DAPI eYFP
-1.7 mm
1
2
4
A1
*
**
A2
#
A4
B
DAPI eYFP
-1.2 mm
1
1'
2
4
5
B1
*
B1'
**
B4
B2
#
B5
C
DAPI eYFP
-0.8 mm
1
1'
2
4
C1
*
C1'
**
C4
C2
##
#
D
DAPI eYFP
-0.3 mm
1
2
4
D1
*
**
D2
##
D4
E
DAPI eYFP
+0.1 mm
2
5
E2
##
E5
F
DAPI eYFP
+0.5 mm
2
5
F2
##
F5

Figure S3

Figures S2 and S3. Widespread axonal projections of the noci-TRAPed elPB neurons, Related to Figure 2.

The eYFP-expressing fibers originating from the elPB in Fig. 2B1 are shown in sequential coronal sections progressing from caudal to rostral planes, sampled from the same nociTRAPed mouse as in Fig. 2B1. These sections are divided into two sets: the caudal portion is presented in Fig. S2, and the rostral portion in Fig. S3. Figure S2 depicts projections in sections ranging from -4.2 to -2.2 mm, and Fig. S3 shows those from -1.7 to +0.5 mm, relative to Bregma.

The eYFP signals (yellow) are overlaid on DAPI-stained images (blue). Rostrocaudal levels (from Bregma): Fig. S2A, -4.2 mm; Fig. S2B, -3.5 mm; Fig. S2C, -2.5 mm; Fig. S2D, -2.2 mm; Fig. S3A, -1.7 mm; Fig. S3B, -1.2 mm; Fig. S3C, -0.8 mm; Fig. S3D, -0.3 mm; Fig. S3E, +0.1 mm; Fig. S3F, +0.5 mm. The panels in the leftmost column with single alphabet (A-D in Fig. S2 and A-F in Fig. S3) indicate low-magnification images of coronal sections arranged vertically from caudal to rostral levels (Fig. S2A, most caudal and Fig. 3F, most rostral; scale bars: 1 mm; the same for images A-D in Fig. S2 and A–F in Fig. S3). The areas surrounded with boxes in these low-magnification images indicate specific subregions where YFP signals were observed and numbered according to the estimated sequence of projection from the nociTRAPed neurons in the right elPB (particularly for boxes 1-4). These areas are displayed at high-magnification in the right columns with alphabet-and-number, with the number corresponding to the boxes in the low-magnification images. All scale bars in the higher-magnification images represent 200 μm.

The fibers originating from the elPB followed three distinct pathways: (1) the dorsal parabrachio-thalamic pathway, (2) the parabrachio-amygdaloid pathway, and (3) the parabrachio-parasubthalamic pathway.

The dorsal parabrachio-thalamic pathway (images with "*"): YFP-expressing fibers passed through the dorsal PAG (Fig. S2A1, Fig. S2B1), heading mediodorsally (Fig. S2C1, Fig. S2D1), and passing through the paraventricular thalamus (PVT; Fig. S3A1, Fig. S3B1, Fig. S3C1), finally arriving at the anterior nucleus of the paraventricular thalamus (PVA; Fig. S3D1). Some fibers branched more ventrally to regions such as the medial preoptic nucleus (MnPO; ** in Fig. S3B1’, Fig. S3C1’, and Fig. S3D1).

The parabrachio-amygdaloid pathway (images with "#"): after passing through the

central tegmental tract (Fig. S2A2, Fig. S2B2, Fig. S2C2), this pathway passed near the lateral hypothalamus (Fig. S2D2, Fig. S3A2, Fig. S3B2), then approached the rostral end of the CeA (Fig. S3C2). Some fibers branched before projecting to the rostral end of the CeA and coursed to the bed nucleus of the stria terminalis (BNST; Fig. S3D2, Fig. S3E2, Fig. S3F2; ##).

We observed a fiber set that first coursed toward the BNST and then took a descending "projection loop" down to the caudal end of the CeA (Fig. S3E2, Fig. S3D4, Fig. S3C4, Fig. S3B4, Fig. S3A4, arriving at the CeA, indicated with white arrowheads), reminiscent of the projection loop of elPB fibers terminating exclusively at the caudal CeC [1].

The parabrachio-parasubthalamic pathway (images with "+"): after passing near the midbrain reticular nucleus (Fig. S2A3, Fig. S2B3), this pathway coursed toward the parasubthalamic nucleus (PSTN) (Fig. S2C3, Fig. S2D3; +), mainly to the PSTN. YFP-positive fibers to the ventral medial hypothalamus and the ventral lateral pre-optic area were limited, unlike vesicular glutamate transporter 2 (VGlut2) positive PB neurons [2]. The dotted lines in Fig. S3B5 and Fig. S3C2 show the border of the amygdala complex, and the dotted line in Fig. S3F2 shows the anterior commissure olfactory limb.

References for Figs. S2 and S3

1. Sarhan, M., Freund-Mercier, M.-J., and Veinante, P. (2005). Branching patterns of parabrachial neurons projecting to the central extended amygdala: Single axonal reconstructions. J Comp Neurol *491*, 418–442. https://doi.org/10.1002/cne.20697.
2. Huang, D., Grady, F.S., Peltekian, L., and Geerling, J.C. (2021). Efferent projections of Vglut2, Foxp2, and Pdyn parabrachial neurons in mice. Journal of Comparative Neurology *529*, 657–693. https://doi.org/10.1002/cne.24975.

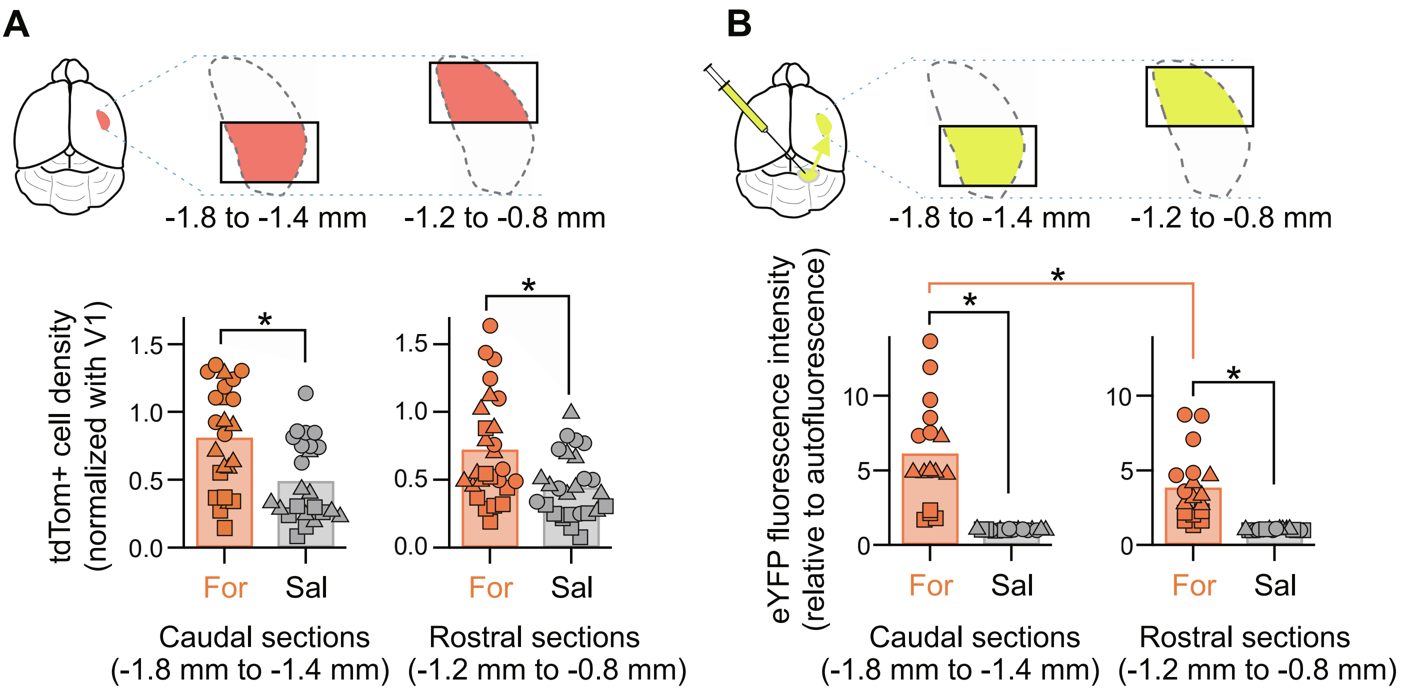
A
-1.8 to -1.4 mm
-1.2 to -0.8 mm
tdTom+ cell density
(normalized with V1)
1.5
1.0
0.5
0.0
*
For
Sal
Caudal sections
(-1.8 mm to -1.4 mm)
Rostral sections
(-1.2 mm to -0.8 mm)
B
eYFP fluorescence intensity
(relative to autofluorescence)
10
5
0

Figure S4

Figure S4. Evaluation of the density of nociTRAPed neurons and the amount of nociTRAPed elPB projections in caudal and rostral CeA of formalin- and saline-TRAPed mice, Related to Figure 3.

A comparison of the relative tdTomato-positive cell density in the CeA (A) and eYFP fluorescence intensity (B) between caudal and rostral sections. The top panels are schemas of the caudal half (-1.8 mm to -1.4 mm from Bregma) and the rostral half (-1.2 mm to -0.8 mm from Bregma) of the CeA, and the bottom panels show the quantifications. Bars represent the average from 18 sections in the caudal and rostral halves of the formalin- or saline-treated groups (6 sections/mouse, 3 mice/group). Circles, triangles, and squares represent the values measured in the CeC, CeL, and CeM (the same data as in the left panels of Fig. 3E and 3F). * with black statistical bars (in S4A and S4B): Mann-Whitney test with Benjamini-Hochberg compensation. $U = 145$, $p = 0.008$, S4A-caudal; $U = 198$, $p = 0.008$, S4A-rostral; $U = 256$, $p < 0.001$, S4B-caudal; $U = 324$, $p < 0.001$, S4B-rostral. * with an orange statistical bar (in S4B): $U = 207$, $p = 0.0372$, Mann-Whitney test with Benjamini-Hochberg compensation.

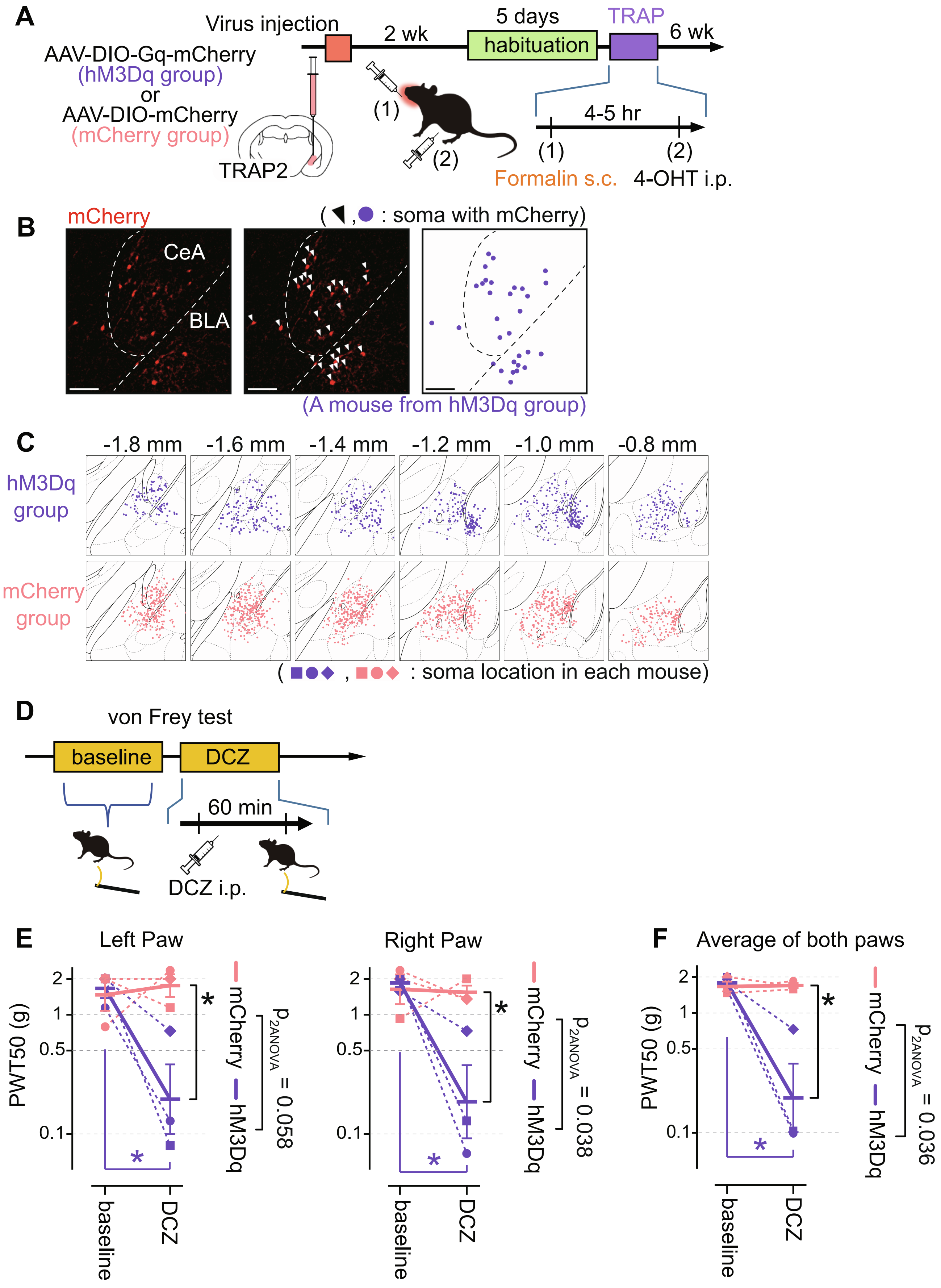
A
Virus injection
2 wk
5 days
habituation
TRAP
6 wk
AAV-DIO-Gq-mCherry
(hM3Dq group)
or
AAV-DIO-mCherry
(mCherry group)
TRAP2
(1)
(2)
4-5 hr
(1)
(2)
Formalin s.c.
4-OHT i.p.
B
mCherry
(, : soma with mCherry)
CeA
BLA
(A mouse from hM3Dq group)
C
-1.8 mm
-1.6 mm
-1.4 mm
-1.2 mm
-1.0 mm
-0.8 mm
hM3Dq group
mCherry group
(, : soma location in each mouse)
D
von Frey test
baseline
DCZ
60 min
DCZ i.p.
E
Left Paw
Right Paw
PWT50 (g)
2
1
0.5
0.1
*
mCherry
hM3Dq
p_{2ANOVA} = 0.058
p_{2ANOVA} = 0.038
baseline
DCZ
F
Average of both paws
p_{2ANOVA} = 0.036

Figure S5

Figure S5. Chemogenetic activation of noci-TRAPed neurons affects paw withdrawal mechanical threshold, Related to Figure 4.

A: Timeline for the TRAP experiment. TRAP2 mice were injected with AAV-DIO-hM3Dq-mCherry (hM3Dq group) or AAV-DIO-mCherry (mCherry group) in the right CeA 3 weeks before orofacial formalin TRAP. Von Frey tests were performed 6 weeks after the TRAPing.

B: Representative images of mCherry expression around CeA region in a mouse from hM3Dq group (-1.8 mm from Bregma). The white arrow heads inidicate detected soma with mCherry expression in the middle panel. The detected soma was plotted with its spatial location (the right panel) and aligned to the atlas images (the next Fig. S5C). Scale bars: 200 µm.

C: All of the detected soma around the CeA region are plotted. Each column shows the coronal level of the brain section (-1.8, -1.6, -1.4, -1.2, -1.0, -0.8 mm from Bregma, left to right). Each row shows hM3Dq group (upper row) and mCherry group (bottom row). Each shaped dot (square, circle, and diamond) represents each mouse (n = 3 in each group).

D: Experimental protocol for the von Frey test. The "baseline" von Frey test was performed the day before the "DCZ" experiment. For the "DCZ" von Frey test, mice were administered deschloroclozapine (DCZ) (100 µg/kg) 60 minutes before the start of the test.

E: Results of the von Frey test at "baseline" and "DCZ". The left and right graphs indicate the paw withdrawal threshold values (PWT50) of the left and right hind paws at baseline and after DCZ administration. Each dotted line with different markers represents values from an individual mouse (hM3Dq (n = 3, blue), mCherry (n = 3, red); same markers represented in Fig. S5C atlas plot). The solid lines indicate the group average. Thresholds are displayed on a log scale. Group factors (mCherry vs. hM3Dq) and time factors (“baseline” vs. “DCZ”) were assessed using a two-way repeated ANOVA. The p values of group factor were shown right next to the graph legend as “p_{2ANOVA}”. The multiple comparisons were performed and adjusted with the Benjamini-Hochberg method. The black statistical bars with * (on the right side of the graphs) indicate a significant difference between the mCherry group and the hM3Dq group at “DCZ”, while dark blue statistical bars with * (on the bottom of the graphs) indicate a significant

difference between the “baseline” and “DCZ” in the hM3Dq group. (Left hind paw, two-way repeated ANOVA; Group factor: p = 0.058, F (1,4) = 6.93; Time factor: p = 0.071, F (1,4) = 5.98; Treatment*Time: p = 0.045, F (1,4) = 8.34. The black * p = 0.005 mCherry vs. hM3Dq in "DCZ". The dark blue * p = 0.0196 “baseline” vs. “DCZ” in the hM3Dq group. Right hind paw, two-way repeated ANOVA; Group factor: p = 0.0379, F (1,4) = 9.328; Time factor: p = 0.056, F (1,4) = 7.054; Treatment*Time: p = 0.0651, F (1,4) = 6.371. The black * p = 0.0049 mCherry vs. hM3Dq in "DCZ". The dark blue * p = 0.0215 “baseline” vs. “DCZ” in the hM3Dq group.)

F: The average of the left and right hind paws. (two-way repeated ANOVA; Group factor: p = 0.036, F (1,4) = 9.576; Time factor: p = 0.030, F (1,4) = 10.80; Treatment*Time: p = 0.0287, F (1,4) = 11.20. The multiple comparisons adjusted with the Benjamini-Hochberg method; The black * p = 0.0019 mCherry vs. hM3Dq in "DCZ"; The dark blue * p = 0.0094 “baseline” vs. “DCZ” in the hM3Dq group.)

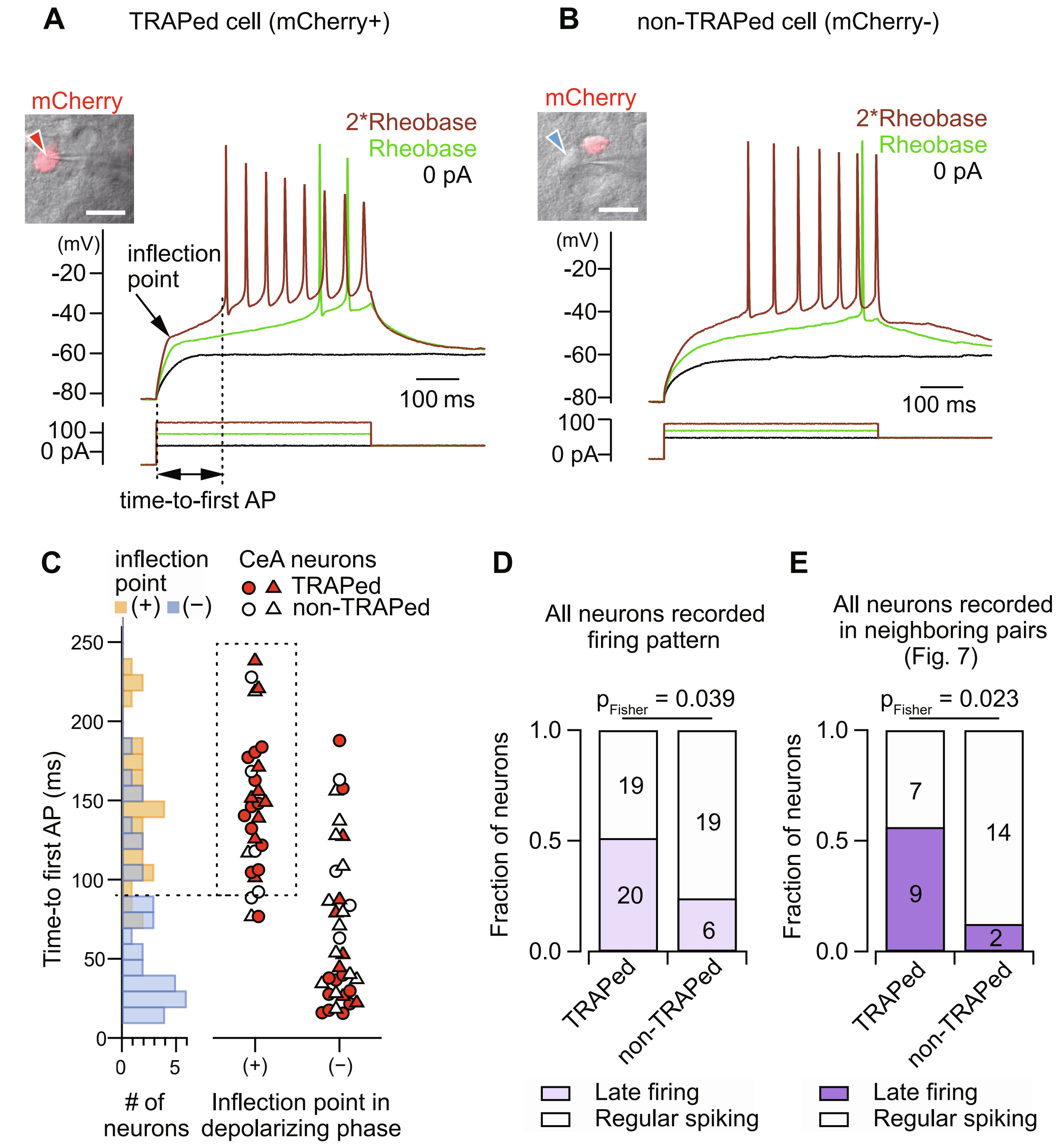

A
TRAPed cell (mCherry+)
mCherry
2*Rheobase
Rheobase
0 pA
(mV)
-20
-40
-60
-80
inflection point
100 ms
100
0 pA
time-to-first AP
B
non-TRAPed cell (mCherry-)
mCherry
2*Rheobase
Rheobase
0 pA
(mV)
-20
-40
-60
-80
100 ms
100
0 pA
C
inflection point
(+) (−)
CeA neurons
TRAPed
non-TRAPed
Time-to first AP (ms)
250
200
150
100
50
0
0
5
# of neurons
(+)
(−)
Inflection point in depolarizing phase
D
All neurons recorded firing pattern
p_Fisher = 0.039
Fraction of neurons
1.0
0.5
0.0
19
20
19
6
TRAPed
non-TRAPed
Late firing
Regular spiking
E
All neurons recorded in neighboring pairs (Fig. 7)
p_Fisher = 0.023
Fraction of neurons
1.0
0.5
0.0
7
9
14
2
TRAPed
non-TRAPed
Late firing
Regular spiking

Figure S6

Figure S6. Firing properties of noci-TRAPed CeA neurons and non-noci-TRAPed CeA neurons, Related to Figure 5.

A, B: Example traces from TRAPed cells (A) and non-TRAPed cells (B) and the definition of the criteria for firing pattern classification. Left upper inset in each panel: bright-field image around the tip of the glass pipette showing patch-clamp recordings from mCherry-positive (A, red arrowhead) or mCherry-negative (B, blue arrowhead) cells. Scale bar: 20 μm. The scale of inset images in panels a and b is the same. The membrane potential trace is shown at the top, illustrating the firing pattern, with the current injection trace at the bottom. The black, green, and brown traces represent membrane potentials in response to step pulses of 0 pA, rheobase, and twice the rheobase, respectively. The firing pattern was classified based on time-to-first AP and the presence of an inflection point, as shown in Fig. S6A. See the details in the Methods and Fig. S6C. Figs. S6A and S6B are examples of late-firing and regular-spiking neurons, respectively. Inflection point: A clear inflection point was observed in the membrane potential trace at twice the rheobase (brown trace in S6A).

C: All CeA neurons were recorded for their firing pattern. Left histogram: Time-to-first AP under a depolarizing pulse at twice the rheobase for all recorded neurons (see Fig. S6A). Right: Each marker indicates the time to the first AP for each cell, with (+) or without (-) an inflection point. Each red-filled marker indicates TRAPed neuron (39 neurons), while each white-filled marker indicates a non-TRAPed neuron (25 neurons). We classified late-firing neurons as those having a delay of more than 90 ms (illustrated with a dashed line) with a clear inflection point. Neurons that did not meet these criteria were classified as having a regular spiking pattern. Triangular and round shapes indicate neurons recorded from caudal and rostral slices of the CeA, respectively.

D, E: Fraction of late-firing neurons (illustrated in purple) in TRAPed and non-TRAPed neurons, calculated from all recorded neurons (D) and neighboring-paired cells (E). Numbers in the bar graph represent the number of neurons. (Odds ratio, 0.31 for Fig. S6D, 0.11 for Fig. S6E; Fisher’s exact test).

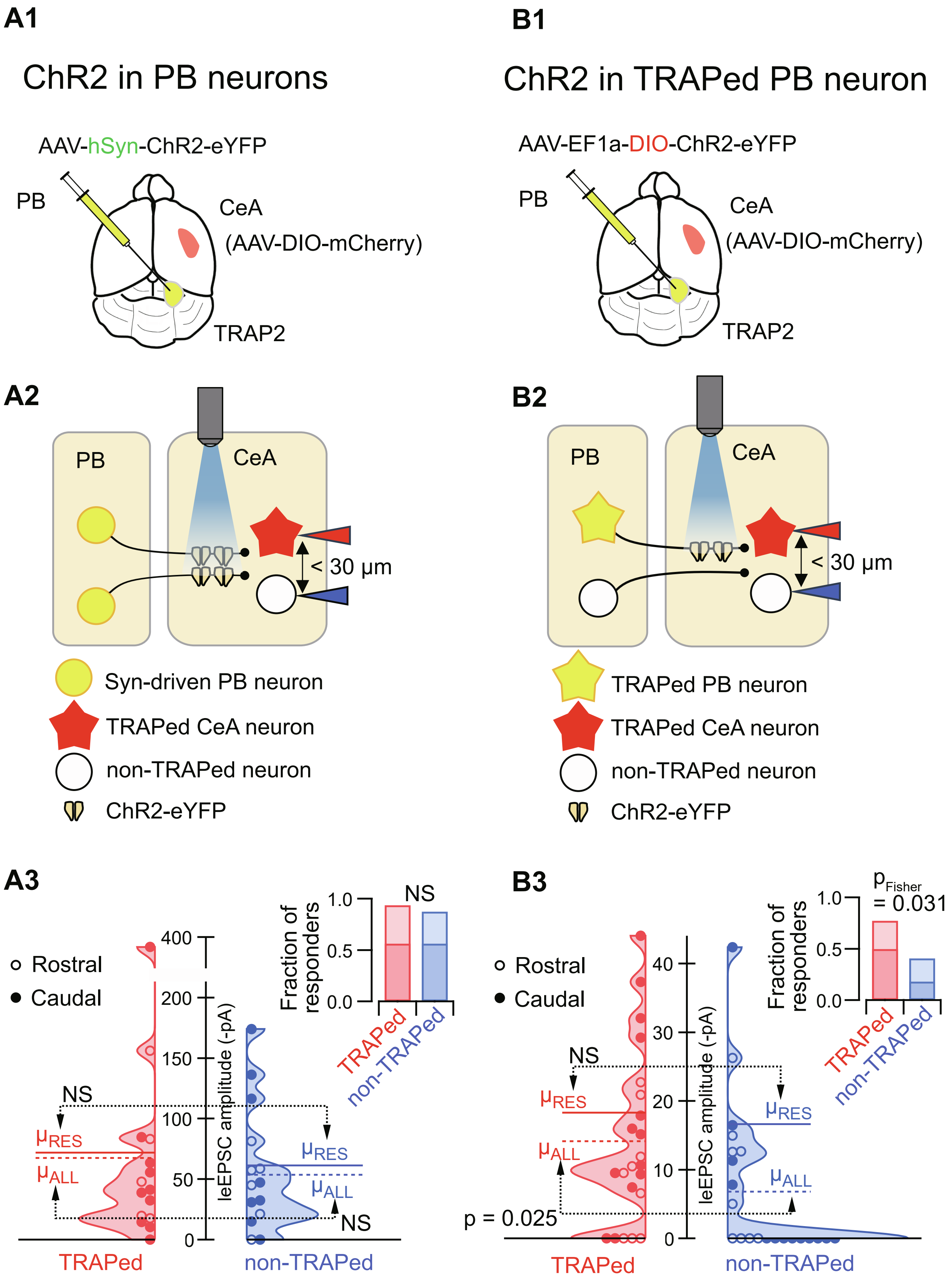
A1
ChR2 in PB neurons
AAV-hSyn-ChR2-eYFP
PB
CeA
(AAV-DIO-mCherry)
TRAP2
B1
ChR2 in TRAPed PB neuron
AAV-EF1a-DIO-ChR2-eYFP
PB
CeA
(AAV-DIO-mCherry)
TRAP2
A2
PB
CeA
< 30 μm
Syn-driven PB neuron
TRAPed CeA neuron
non-TRAPed neuron
ChR2-eYFP
B2
PB
CeA
< 30 μm
TRAPed PB neuron
TRAPed CeA neuron
non-TRAPed neuron
ChR2-eYFP
A3
Rostral
Caudal
NS
μRES
μALL
leEPSC amplitude (-pA)
400
200
150
100
50
0
NS
TRAPed
non-TRAPed
Fraction of responders
1.0
0.5
0.0
NS
TRAPed
non-TRAPed
B3
Rostral
Caudal
NS
μRES
μALL
p = 0.025
leEPSC amplitude (-pA)
40
30
20
10
0
TRAPed
non-TRAPed
Fraction of responders
pFisher = 0.031
1.0
0.5
0.0
TRAPed
non-TRAPed

Figure S7

Figure S7. Comparison of excitatory synaptic transmission between general/noci-TRAPed PB neuron terminals and noci-TRAPed/non-TRAPed CeA neurons, Related to Figure 6.

A, B: Synaptic responses induced by light stimulation of terminals from general PB neuron population (A1-A3, same as "syn-driven elPB" in Fig. 6B1) and noci-TRAPed PB (B1-B3, same as "TRAPed elPB" in Fig. 6B2) in all neighboring pairs of recorded neurons in the CeA along the rostrocaudal axis. AAV-hSyn-DIO-mCherry was injected into the CeA in both experimental conditions as in Fig. 6. **A1**: AAV-hSyn-ChR2-eYFP was injected into the PB. **B1**: AAV-EF1a-DIO-ChR2-eYFP was injected into the PB. **A2, B2**: Schematic illustrations of optogenetic patch-clamp recordings from neighboring pairs of TRAPed (red star) and non-TRAPed (white circle) CeA neurons. Red and blue triangles indicate recording pipettes targeting the noci-TRAPed and non-TRAPed CeA neurons, respectively. **A2**: ChR2-eYFP is expressed in a Cre-independent manner, activating non-selective elPB axons upon blue light stimulation. **B2**: ChR2-eYFP is expressed only in noci-TRAPed elPB neurons, activating only noci-TRAPed elPB axons upon blue light stimulation. **A3, B3**: Violin plots displaying the amplitude of light-evoked EPSCs from TRAPed neurons (red) and non-TRAPed neurons (blue). Open and filled circles indicate EPSC amplitude recorded from rostral and caudal CeA, respectively. Colored dashed horizontal lines indicate the mean amplitude of all recorded neurons (μ_{ALL}: A3: TRAPed vs. non-TRAPed neurons, 66.8 pA vs. 53.5 pA, n = 16 in each, U = 127, p = 0.98, Mann-Whitney test; B3: TRAPed vs. non-TRAPed neurons, 14.12 pA vs. 6.79 pA, n = 22 in each, U = 334.5, p = 0.025, Mann-Whitney test). Colored thick horizontal lines represent the mean amplitude of responding neurons (μ_{RES}: A3: 70.5 pA vs. 60.6 pA, n = 15 and 14, respectively, U = 96, p = 0.71, Mann-Whitney test; B3: 18.3 pA vs. 16.6 pA, n = 17 and 9, respectively, U = 81, p = 0.83, Mann-Whitney test). Inset stacked bar graphs show the fraction of responsive neurons (A3: 15/16 TRAPed neurons vs. 14/16 non-TRAPed neurons, p > 0.99; odds ratio, 0.48; Fisher's exact test; B3: 17/22 TRAPed neurons vs. 9/22 non-TRAPed neurons, p = 0.031; odds ratio, 0.21; Fisher's exact test). Dark-shaded bars indicate caudal neurons, while light-shaded bars indicate rostral neurons.

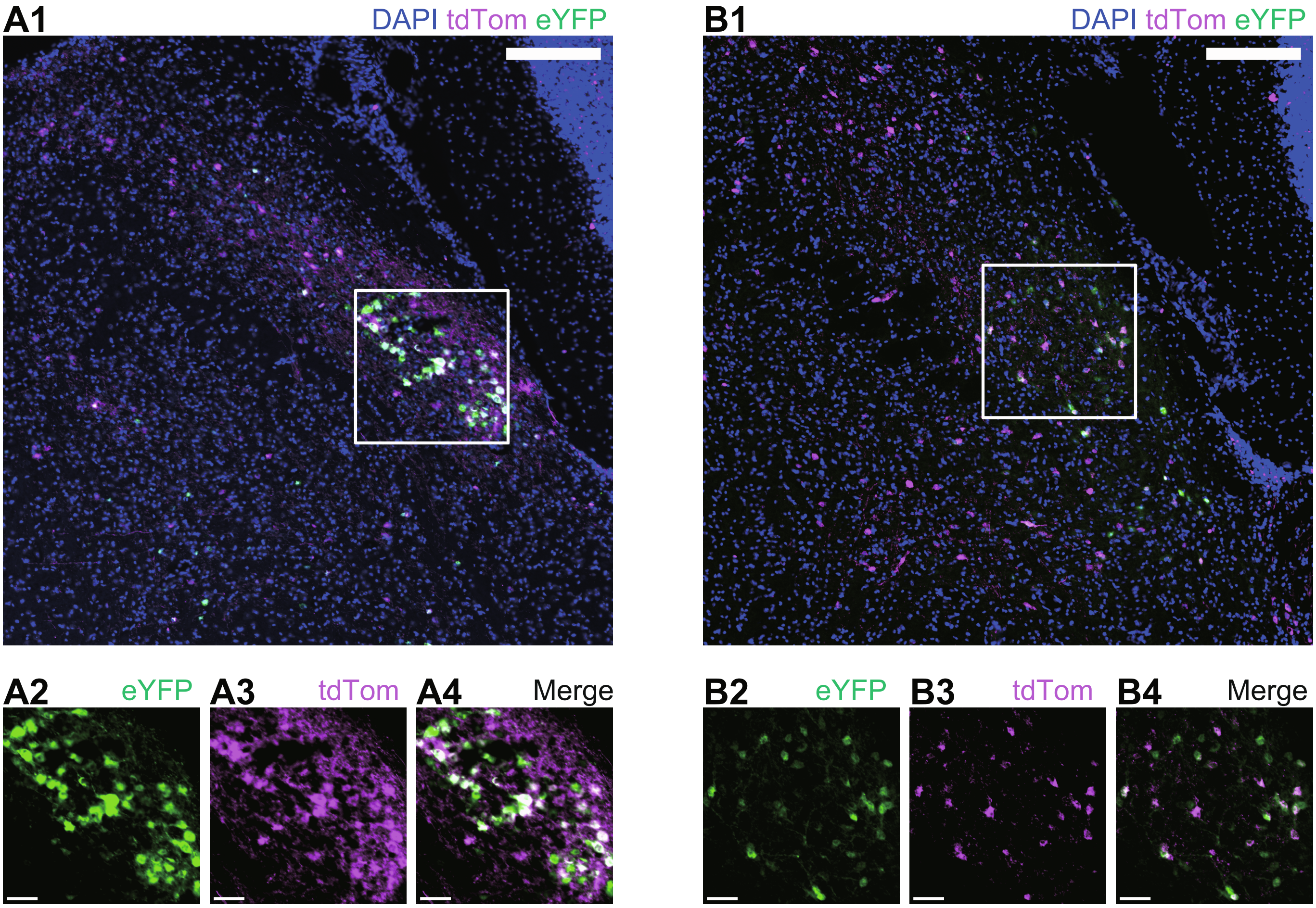
A1
DAPI tdTom eYFP
B1
DAPI tdTom eYFP
A2
eYFP
A3
tdTom
A4
Merge
B2
eYFP
B3
tdTom
B4
Merge

Figure S8

Figure S8. Color-compatible version of Figure 2c1 and 2c2, Related to Methods.

A1 and **B1** correspond to Figure 2C1 and Figure 2C2, respectively. Blue: DAPI; magenta: tdTomato; green: eYFP. Scale bars: 200 μm (approximately -5.4 mm from Bregma).

A2 and **B2** show the eYFP signal, while **A3** and **B3** display the tdTomato signal from the boxed areas in A1 and B1, respectively. Scale bars: 50 μm.

A4 shows the merged image of A2 and A3. **B4** shows the merged image of B2 and B3. Scale bars: 50 μm.
